# p180 Promotes the Ribosome-Independent Localization of a Subset of mRNA to the Endoplasmic Reticulum

**DOI:** 10.1371/journal.pbio.1001336

**Published:** 2012-05-29

**Authors:** Xianying A. Cui, Hui Zhang, Alexander F. Palazzo

**Affiliations:** Department of Biochemistry, University of Toronto, Toronto, Ontario, Canada; Medical University of Vienna, Austria

## Abstract

In metazoans, the majority of mRNAs coding for secreted and membrane-bound proteins are translated on the surface of the endoplasmic reticulum (ER). Although the targeting of these transcripts to the surface of the ER can be mediated by the translation of a signal sequence and their maintenance is mediated by interactions between the ribosome and the translocon, it is becoming increasingly clear that additional ER-localization pathways exist. Here we demonstrate that many of these mRNAs can be targeted to, and remain associated with, the ER independently of ribosomes and translation. Using a mass spectrometry analysis of proteins that associate with ER-bound polysomes, we identified putative mRNA receptors that may mediate this alternative mechanism, including p180, an abundant, positively charged membrane-bound protein. We demonstrate that p180 over-expression can enhance the association of generic mRNAs with the ER. We then show that p180 contains a lysine-rich region that can directly interact with RNA in vitro. Finally, we demonstrate that p180 is required for the efficient ER-anchoring of bulk poly(A) and of certain transcripts, such as *placental alkaline phosphatase* and *calreticulin*, to the ER. In summary, we provide, to our knowledge, the first mechanistic details for an alternative pathway to target and maintain mRNA at the ER. It is likely that this alternative pathway not only enhances the fidelity of protein sorting, but also localizes mRNAs to various subdomains of the ER and thus contributes to cellular organization.

## Abstract 2

The localization of many secretory mRNAs to the endoplasmic reticulum does not require ribosomes or translation, but is instead promoted by p180, an abundant, membrane-bound protein that likely binds directly to mRNA.

## Author Summary

Messenger RNAs (mRNAs) that encode secreted or membrane-bound proteins must be delivered to, and then maintained on, the surface of the endoplasmic reticulum (ER). These mRNAs encode a short polypeptide that targets the mRNA/ribosome/nascent protein complexes to the ER surface during translation; however, recent studies support the existence of additional ER-localization signals that might be present within the mRNA molecules themselves. Here, we demonstrate that a fraction of these mRNAs, whose encoded proteins are destined for secretion, contain information that targets and anchors them to the ER independently of their encoded polypeptide or their association to ribosomes. We identify proteins on the ER that may serve as receptors for these mRNAs. We then show that one of these candidate membrane-bound receptors, p180, is required for the maintenance of certain mRNAs on the surface of the ER even after their translation into protein is disrupted. We also demonstrate that p180 contains a region that binds directly to RNA and likely mediates the anchoring of mRNA to the ER. Our study thus provides the first mechanistic details of an alternative pathway used to ensure that secretory mRNAs, and their encoded proteins, reach their proper destination in the ER.

## Introduction

The localization of mRNAs to various subcellular sites, through the interaction of transcripts with mRNA localization proteins, is a widespread phenomenon important for the proper sorting of proteins to their final destination, and for the fine tuning of gene expression to the local requirements of a subcellular region. A systematic analysis of the distribution of mRNAs in the *Drosophila melanogaster* embryo indicates that transcripts from approximately 70% of the protein-coding genes localized to particular subcellular regions [1].

One major class of transcripts, those encoding membrane and secreted proteins, are targeted to and translated on the endoplasmic reticulum (ER). While the ER is one continuous membrane system that is distributed throughout the cell, even transcripts translated on this organelle can be distributed asymmetrically. One prominent example is the localization of the *wingless* transcript to the apical cytoplasm of *Drosophila* ectodermal cells, which is crucial to embryonic development [2]. In a variety of other polarized systems, including *Xenopus* oocytes [3], plant endosperm cells [4], and budding yeast [5], asymmetrically localized mRNAs have been reported to use the ER as a scaffold. How mRNAs can be localized to distinct ER locales, however, still remains largely unknown.

Presumably, subsets of mRNAs that share a common subcellular distribution should bind to a common RNA receptor. This idea is supported by two large-scale analyses which demonstrated that each RNA-binding protein in *Saccharomyces cerevisiae* tends to associate with transcripts encoding functionally related proteins [6],[7]. These associations may help to localize certain classes of mRNAs to different organelles. For example, 90% of the transcripts associated with the pumilio protein, Puf3p, code for mitochondrial proteins in budding yeast [6]. Puf3p localizes to mitochondria [8] and is required for the targeting of many of these mRNAs to this organelle [9],[10]. Several other RNA-binding proteins have been shown to preferentially associate with mRNAs encoding secreted or membrane-bound proteins in yeast [7],[11],[12]. It remains unclear, however, whether these interactions function to localize mRNAs to the ER.

The only conserved mechanism identified thus far for localizing mRNAs to the ER is through the canonical signal sequence directed pathway. This targeting process is initiated during the translation of mRNAs encoding secreted and membrane-bound proteins, when a nascent N-terminal signal sequence or transmembrane segment recruits the signal recognition particle (SRP) to the translating ribosome [13]. Subsequent interactions between SRP and an ER-bound SRP receptor promote the re-localization of the mRNA/ribosome/nascent polypeptide chain complex to the surface of the ER [14]. After targeting is complete, the signal sequence or transmembrane segment is transferred to the protein-conducting channel formed by the Sec61 translocon complex [15] and the mRNA is retained on the surface of the ER by direct interactions of the translating ribosome with this channel [16]. Despite all the intensive work performed on the secretory pathway, it remained unclear until very recently whether additional ribosomal-independent interactions exist between these mRNAs and putative RNA receptors on the ER.

Classic cell fractionation studies have provided evidence both for [17]–[20] and against [21],[22] ribosome-independent interactions. More recent studies have provided data that support the existence of an alternative mRNA targeting pathway. For example, certain mRNAs remain associated with ER-derived microsomes even after ribosomes are partially stripped off [23],[24]. Moreover, mRNAs that encode cytoplasmic polypeptides have also been found to bind to microsomes [23]–[26]. Furthermore, mRNAs remained ER-associated in HeLa cells that are depleted of SRP54, an essential component of the SRP [24]. Despite all these observations, it remains possible that alternative polypeptide-based targeting pathways exist that recognize other features in the newly synthesized protein besides the signal sequence. For example, in vertebrates, the Sec62/Sec63 complex and the ERj1 protein, which have both chaperone and ribosome binding domains facing the cytoplasm, might serve to anchor translating ribosomes to the surface of the ER independently of the signal sequence and the SRP system [27]–[29].

Here we provide conclusive evidence that mRNAs are targeted and retained on the surface of the ER independent of translation and ribosomes. We also provide, to our knowledge, the first mechanistic details on this alternative ER-localization pathway. In particular we demonstrate that p180, an abundant membrane-bound protein that co-fractionates with ER-derived mRNA, promotes the general association of mRNA with the surface of the ER membrane. This activity is likely mediated in part by a lysine-rich region in this ER-resident protein that can directly interact with RNA in vitro. Finally, we show that p180 is required for the ER-anchoring of certain transcripts. We thus shed light on the workings of a basic biological process that up until now remained poorly characterized and underappreciated.

## Results

### ER-Targeted Transcripts and Ribosomes Co-Localize with the ER in Digitonin-Extracted Cells

Although the ribosome-independent association of mRNA to the ER has been extensively examined using cell fractionation, these measurements require the interaction between ribosome-free transcripts and ER-derived microsomes to be stable over long time intervals outside of the cellular context. Ideally one could overcome these problems by investigating the ER-association of poly(A) transcripts within the cellular environment. To overcome these potential problems we investigated the ER-association of poly(A) transcripts within the cellular environment using microscopic analysis. In order to visualize ER-bound poly(A) mRNA, mammalian tissue culture cells were first treated with low levels of digitonin to selectively permeabilize the plasma membrane, thereby extracting the cytoplasm and all unbound transcripts while maintaining the integrity of the ER membrane [25]. In order to ensure that this technique effectively separates these two classes of mRNA while simultaneously preserving the ultrastructure of the ER, the distribution of various versions of the *fushi tarazu* (*ftz*) mRNA fragment were examined in COS-7 cells by fluorescent in situ hybridization (FISH). First we monitored *t-ftz* mRNA, which encodes a secreted version of the ftz protein [30]. This mRNA, which localizes to the ER ([30] and Figure S1A), remained associated with the cells after digitonin extraction (Figure S1B–C). Next we monitored *c-ftz-i* mRNA, which encodes a soluble, cytoplasmic version of the ftz polypeptide. The majority of this transcript distributed diffusely across the cytoplasm in intact cells (Figure S1D) and was extracted when cells were treated with digitonin (Figure S1E). Note that nuclear *t-ftz* and *c-ftz-i* transcripts were resistant to extraction since digitonin treatment does not permeabilize the nuclear envelope [31]. We also monitored the distribution of the soluble adenosine kinase (AdK) enzyme by indirect immunofluorescence. As previously reported [32], this protein was present in both the nucleus and cytoplasm in intact COS-7 cells (Figure S1F). However, after digitonin treatment only the nuclear fraction remained (Figure S1G). Finally, we monitored the distributions of ribosomes and Trapα, a membrane-bound ER protein that associates with the Sec61 translocon [33]. The cellular distribution of Trapα was largely unaffected by extraction, indicating that digitonin extraction did not disrupt the integrity of the ER. In contrast, the large ribosomal protein RPLP0 localized in a diffuse cytoplasmic pattern in intact cells (Figure S1H) and in a reticular pattern that co-localized with Trapα in digitonin-extracted cells (Figure S1I). In summary, digitonin extraction effectively removes cytoplasmic, but not nuclear or ER-associated factors, while simultaneously preserving ER-morphology.

### mRNA Remains Associated to the ER Independently of Translation and Ribosome-Association

**Figure 1:**
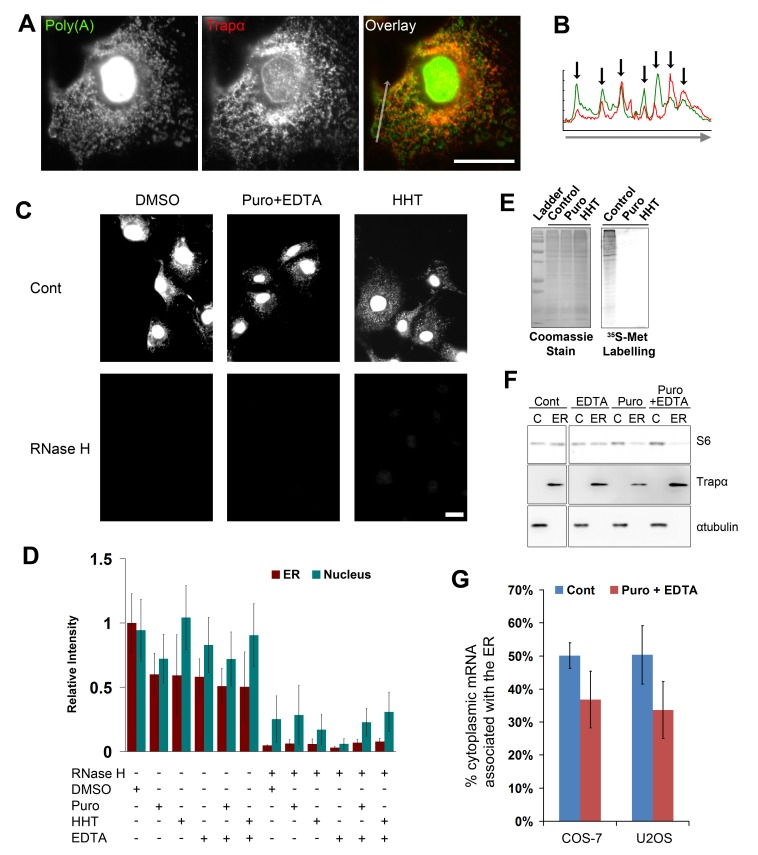
Poly(A) transcripts associate with ER independently of ribosomes and translation. (A) A single digitonin-extracted COS-7 cell co-stained for poly(A) mRNA using poly(dT) FISH probes, and for the ER marker Trapα by immunofluorescence. Note the general co-localization between the mRNA (green) and Trapα (red). (B) The fluorescence intensity (*y*-axis) of the poly(A) mRNA (green) and Trapα (red) along the arrow (*x*-axis) in the overlay image in (A). Note the correlation between peaks in intensity of poly(A) mRNA and Trapα (black arrows). (C–D) COS-7 cells were treated with either DMSO, puromycin (“Puro”), homoharringtonine (“HHT”) for 30 min, and then extracted with digitonin alone or with 20 mM EDTA. Cells were then fixed, stained for poly(A) mRNA using poly(dT) FISH probes, and then treated with RNase H (RNase H “+”) or control buffer (“Cont” or RNase H “−”) for 1 h at 37°C. Cells were imaged (C) and the fluorescence intensity of the ER and nucleus were quantified (D). Each bar represents the average and standard error of three independent experiments, each consisting of the average integrated intensity of 30 cells over background normalized to the signal in the ER of DMSO/control treated cells. Note that in cells not treated with RNase H, the amount of mRNA bound to the ER decreased by about half in all the drug-treated cells as compared to DMSO-treated cells. In contrast RNase H treatment eliminated most of the ER fluorescence and the majority of the nuclear signal. All scale bars = 20 µm. (E) COS-7 cells were treated with control medium (DMSO), puromycin, or HHT for 15 min, then incubated in ^35^S-methionine to label newly synthesized proteins for an additional 15 min. Cell lysates were collected and separated by SDS-PAGE. Total proteins were visualized by Coomassie blue stain, and newly synthesized proteins were detected by autoradiography. Molecular weight markers are indicated on the left (“Ladder”; 188 kD, 62 kD, 49 kD, 38 kD, 28 kD, 18 kD). (F) COS-7 cells were either treated with DMSO (“Cont”) or puromycin for 30 min, then extracted with digitonin (in the absence or presence of 20 mM EDTA). Cytoplasmic (“C”; i.e., non-ER) and ER (“ER”) fractions were separated by SDS-PAGE, then transferred to nitrocellulose, and immunoblotted with antibodies against the small ribosomal protein S6, the ER marker Trapα, and the cytoplasmic marker αtubulin. Note that most of the S6 protein is released from the membrane to the cytoplasmic fraction only after cells are treated with puromycin and then extracted with EDTA. (G) COS-7 and U2OS cells were treated either with cyclohexamide (“control”) and then extracted, or with puromycin for 30 min and then extracted in the presence of 20 mM EDTA. ER and cytoplasmic fractions were isolated as in (F) except that either cyclohexamide, or puromycin and EDTA, was present in all solutions. cDNA was synthesized from each fraction using poly(dT) primers and ^32^P-dNTPs, and ratio of counts in the ER to total (cytoplasm+ER) were tabulated. Each bar represents the average and standard error of three independent experiments.

Having validated a procedure to visually isolate ER-bound molecules, the distribution of ER-associated poly(A) transcripts was then analyzed. We performed FISH on digitonin extracted COS-7 cells with fluorescently labeled poly(dT) oligonucleotides and found a substantial amount of fluorescence in the cytoplasm that co-localized with the ER marker Trapα (Figure 1A). This co-localization was verified by analyzing line scans of the respective fluorescent intensities across the same region of the cell (Figure 1B). Interestingly, although the overall distribution was similar (Figure 1B, black arrows), different regions of the ER were enriched in either poly(A) or Trapα (Figure 1B, note the relative levels of the two markers at each black arrow). To ensure that the FISH signal was caused by an association of our probes with mRNA, we treated the hybridized cells with RNase H, an enzyme that specifically degrades RNA that is hybridized to DNA. Indeed, this treatment dramatically reduced the FISH signal when compared to samples exposed to control buffer (Figure 1C, compare “Cont” to “RNase H”; see Figure 1D for quantification). From these results, we conclude that the staining observed with fluorescent poly(dT) oligonucleotide in digitonin-treated cells represented ER-bound poly(A) transcripts.

Next, the ribosome-independent retention of mRNAs on the ER was assessed. First, poly(A) transcripts were visualized in cells treated with homoharringtonine (HHT), a compound that prevents the initiation of translation while allowing engaged ribosomes to complete translation and naturally fall off the transcript [34]. About half of the mRNA remained associated with the ER after cells were treated with HHT for 30 min (Figure 1C–D), despite the fact that all detectable translation was inhibited after 15 min of treatment, as assayed by the incorporation of ^35^S-methionine into newly synthesized proteins (Figure 1E). Again, the incubation of HHT-treated cells with RNase H eliminated the fluorescence signal (Figure 1C–D). Next, mRNA was visualized in cells treated with the translation inhibitor puromycin. This compound ejects the nascent polypeptide chain from the ribosome, facilitating the dissociation of small and large ribosomal subunits. After dissociation, the large subunit will remain bound to the Sec61 channel, while the small subunit is released from the membrane [35],[36]. To further disrupt ribosomes, EDTA was included in the digitonin extraction buffer to chelate magnesium, which is required for the subunits to remain bound to each other. In order to monitor the release of the small ribosomal subunit, we probed ER and cytoplasmic (i.e., non-ER) fractions with antibodies directed against the small ribosomal protein S6. In untreated COS-7 cells, about half of all small ribosomal subunits were associated with ER membranes (Figure 1F, compare non-ER cytoplasm “C” with ER membranes “ER” in the control “Cont” cell fractions). However when cells were treated with puromycin for 30 min and then extracted in the presence of EDTA, small ribosomal subunits were efficiently removed from the ER (Figure 1F, “Puro+EDTA”). Note the incomplete removal of small subunits when cells were treated with EDTA or puromycin alone. We then monitored the distribution of mRNA in these cells. In agreement with our previous results, we found that approximately half of all mRNA remained associated to the ER after ribosomes were disrupted by puromycin and EDTA (Figure 1C–D). Again the FISH signal was reduced after RNase H treatment (Figure 1C–D). We also observed that mRNA was retained on the ER in a human osteosarcoma cell line (U2OS) treated with either HHT or a combination of puromycin and EDTA, as analyzed by poly(A) staining (unpublished data).

To further confirm these results, we biochemically analyzed the poly(A) RNA content in subcellular fractions that were prepared from cells treated with either cyclohexamide, a translation inhibitor that stabilizes polysomes, or puromycin and EDTA. We then converted mRNA isolated from cytoplasmic and ER cell fractions into cDNA using poly(dT) primers and radiolabeled nucleotides and then quantified the radioactivity incorporated in each library. We found that in both cyclohexamide-treated COS-7 and U2OS cells, about 50% of the non-nuclear RNA was associated with the ER (Figure 1G, “Cont”) and that this fraction dropped to about 35% after puromycin/EDTA treatment (Figure 1G, “Puro+EDTA”).

From these results, we concluded that a substantial fraction of ER-anchored transcripts are maintained on the ER independently of ribosomes in various mammalian tissue culture cell lines.

### The Extent of ER-Retention After Ribosome Dissociation Differs Among mRNA Species

**Figure 2:**
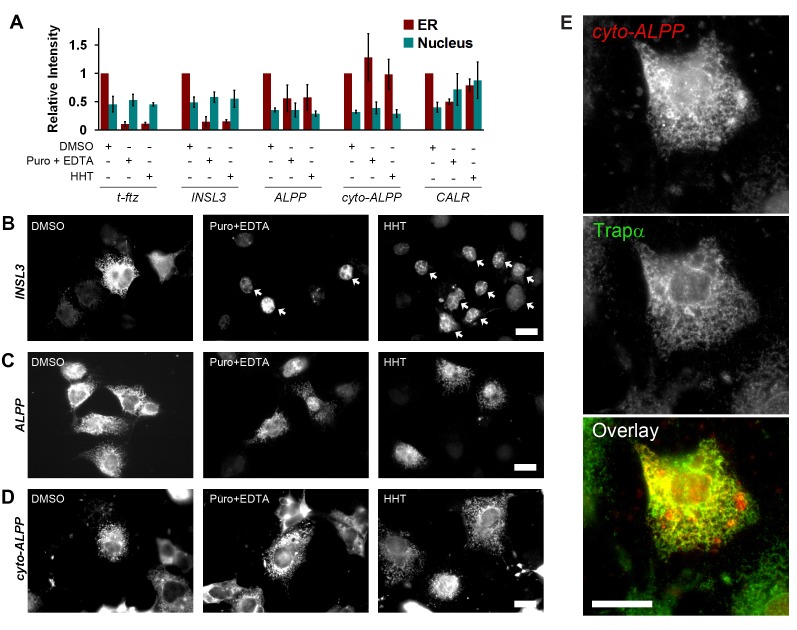
*ALPP* and *CALR*, but not *t-ftz* or *INSL3*, mRNA remain associated with the ER independently of ribosomes and translation. (A–E) COS-7 cells were transfected with plasmids containing either the *t-ftz* (A), *INSL3* (A–B), *ALPP* (A, C), *cyto-ALPP* (a version of *ALPP* lacking signal sequence and transmembrane domain coding regions; A, D–E), or *CALR* (A) genes and allowed to express mRNA for 18–24 h. The cells were then treated with DMSO (“Cont”), puromycin, or HHT for 30 min, and then extracted with digitonin alone or with 20 mM EDTA. Cells were then fixed, stained for mRNA using specific FISH probes, and imaged (see panels B–D for examples). The fluorescence intensities of mRNA in the ER and nucleus in the micrographs were quantified (A). Each bar represents the average and standard error of three independent experiments, each consisting of the average integrated intensity of 30 cells over background. Note that although ribosome disruption caused *INSL3* mRNA to dissociate from the ER, the nuclear mRNA was unaffected (B, nuclei are denoted by arrows). (E) A single field of view containing a single HHT-treated, digitonin-extracted, COS-7 cell expressing *cyto-ALPP* mRNA. *cyto-ALPP* mRNA was visualized by FISH and for Trapα protein by immunofluorescence. Note the extensive co-localization of *cyto-ALPP* mRNA (red) and Trapα (green) in the overlay. All scale bars = 20 µm.

Next, the distribution of transcripts from individual genes was monitored by conventional FISH in COS-7 cells. The majority of these genes have a signal sequence coding region (SSCR), which not only encodes ER-targeting polypeptides but also contains an RNA element that promotes nuclear export and the proper cytoplasmic localization of transcripts [30],[37]. With this in mind, we first investigated the cellular distribution of the reporter transcript *t-ftz*, which contains the SSCR from a mouse Major Histocompatibility Complex (MHC) H2kb gene [30]. Besides the SSCR, this artificial transcript does not contain any sequence that is normally associated with the ER, as it was derived from a transcription factor gene from *Drosophila* [38]. Interestingly, *t-ftz* mRNA, which localizes to the ER in extracted cells (Figure S1A), no longer associated with this organelle after ribosome disruption using either HHT or puromycin/EDTA (Figure 2A, Figure S2A). Note that the amount of nuclear *t-ftz* transcript was unaltered by any of the treatments (Figure 2A), indicating that the change in ER-associated fluorescence was not due to changes in expression levels or FISH efficiency. Thus, we conclude that although the MHC SSCR can promote nuclear export, it is not sufficient to allow mRNAs to be maintained on the surface of the ER after ribosome dissociation.

To determine whether natural mRNAs are maintained on the ER independently of ribosomes, the distribution of transcripts generated from transfected plasmids containing the *insulin-like 3* (*INSL3*), *placental alkaline phosphatase* (*ALPP*), or *calreticulin* (*CALR*) genes was monitored in extracted cells. Previously it had been demonstrated that *CALR* mRNA co-fractionated with microsomes in cells with inactivated SRP and partly remained associated to microsomes after they were partially stripped of ribosomes [24]. Although the association between *INSL3* mRNA and the ER was abolished by either HHT or puromycin/EDTA treatment (Figure 2A–B), about half of the *ALPP* and *CALR* transcripts remained ER-associated under similar conditions (Figure 2A,C, Figure S2B). As seen previously with the *t-ftz* transcript, the amount of nuclear *INSL3* mRNA was unaffected by either HHT or puromycin/EDTA treatments (see arrows in 2B, for quantification see Figure 2A). Interestingly the amount of nuclear *CALR* mRNA increased after the inhibition of translation, although this was quite variable (Figure 2A). This increase is likely attributable to a slight block in nuclear export experienced by certain mRNAs after translation inhibition, as we previously reported [30]. The retention of *ALPP* mRNA on the ER after HHT-treatment was confirmed by the co-localization of these transcripts with Trapα in digitonin-extracted cells (Figure S2C). To further validate our findings, we repeated these experiments with pactamycin, another inhibitor of translation initiation. Pactamycin also allows ribosomes to naturally fall off the transcript while preventing new ribosomes from associating with the mRNA [39]. Indeed, pactamycin effectively inhibits all protein production within 15 min (Figure S3A) and disrupts the ER-localization of *t-ftz* mRNA (Figure S3B), as seen previously [30]. Moreover, in agreement with our other findings, pactamycin treatment disrupts the ER-localization of *INSL3* but not *ALPP* mRNA (Figure S3C–D).

To eliminate the possibility, however remote, that the ER-association of *ALPP* mRNA is signal-sequence dependent, we generated a new construct where the signal sequence and transmembrane domain coding regions of this gene were eliminated. To ensure that the newly synthesized mRNA was efficiently exported from the nucleus, we inserted the frame shifted SSCR from MHC to the 5′end of the truncated *ALPP* ORF. Previously we demonstrated that frame shifted MHC SSCR, which encodes a soluble cytoplasmic (i.e., not ER-targeted) polypeptide, promotes efficient nuclear mRNA export but not ER-targeting of either the mRNA or the encoded protein [30]. We expressed this new fusion construct (*cyto-ALPP*) in COS-7 cells and analyzed its association to the ER. Indeed this mRNA was efficiently retained on the ER after extraction (Figure 2A,D) despite the fact that the encoded protein lacked any features that would target it for secretion. Strikingly, the level of ER-association for *cyto-ALPP* mRNA was unaffected by HHT or puromycin/EDTA treatments (Figure 2A,D). This result further supports the notion that the localization of this transcript to the ER was completely independent of translation. Moreover, the association of *cyto-ALPP* mRNA to the ER after HHT treatment was validated by the co-localization of these transcripts with Trapα in digitonin-extracted cells (Figure 2E). Thus we concluded that the ER-association of *ALPP* mRNA to the ER was independent of features within the encoded polypeptide that are recognized by the SRP targeting pathway.

**Figure 3:**
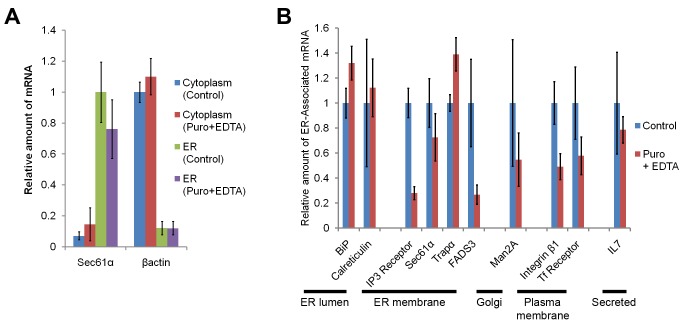
Endogenous mRNAs remain associated with the ER independently of ribosomes and translation. (A) The levels of *Sec61α* and *βactin* transcripts isolated from unbound (i.e., non-ER) cytoplasmic and ER fractions from either cycloheximide treated (“Control”) or puromycin treated EDTA extracted (“Puromycin+EDTA”) U2OS cells were assessed by quantitative RT-PCR analysis. Each bar represents the levels of the specified transcript normalized to 28S rRNA levels, standardized to the level of mRNA in the control sample, and averaged between three independent experiments. Error bars represent the standard error of the mean. Note that 28S rRNA was used as the large ribosomal subunit is known to associate to the ER even after puromycin treatment [35] and that ribosomes are equally distributed in cytoplasmic and ER (see Figure 1F). The level of *Sec61α* mRNA was normalized to the ER fraction from control cells, while the *βactin* mRNA was normalized to the cytoplasmic fraction from control cells. (B) The levels of several transcripts in the ER fraction were analyzed as in (A). Measured transcripts include those encoding ER luminal proteins (BiP, Calreticulin), ER membrane proteins (Inositol-3-phosphate Receptor (IP3 Receptor), Sec61α, Trapα, and Fatty Acid Desaturase 3 (FADS3)), a Golgi protein (Mannosidase 2A (Man2A)), plasma membrane proteins (Integrin β1, and Transferrin Receptor (Tf Receptor)), and a secreted protein (Interleukin 7 (IL7)). All measurements were standardized to the level of mRNA in the ER fraction from control cells.

To determine whether endogenous mRNAs also displayed this activity, we analyzed the level of 10 different transcripts in ER and cytoplasmic fractions (see Figure 1G) using quantitative reverse-transcription PCR. To ensure that our fractionation protocol separated ER-bound transcripts from the rest, we first analyzed the distribution of *Sec61α* and *βactin* mRNAs. The first mRNA encodes the central component of the translocon and was predominantly found in the ER fraction, even when the fractions were derived from cells treated with puromycin and EDTA (Figure 3A). In contrast most of the *βactin* mRNA was in the cytoplasmic fraction. We then extended these studies to transcripts encoding ER-resident, Golgi, plasma membrane, and secreted proteins. The majority of these mRNAs remained in the ER fraction even after puromycin/EDTA treatments (Figure 3B). As with over-expressed *CALR*, endogenous *CALR* mRNA was retained on the ER to a high degree after puromycin/EDTA treatment. Again this activity varied between different transcripts; for example, mRNAs encoding the Inositol-3-Phosphate Receptor (IP3 Receptor) and Fatty Acid Desaturase 3 proteins (FADS3) exhibited a greater dependency on translation than other tested transcripts.

From these results we concluded that mRNAs from the majority of genes that encode secreted or membrane-bound proteins are retained on the ER in a manner that does not require translation or ribosome-association.

### *ALPP* and *CALR* mRNAs Partially Target to the ER Independently of Translation

**Figure 4:**
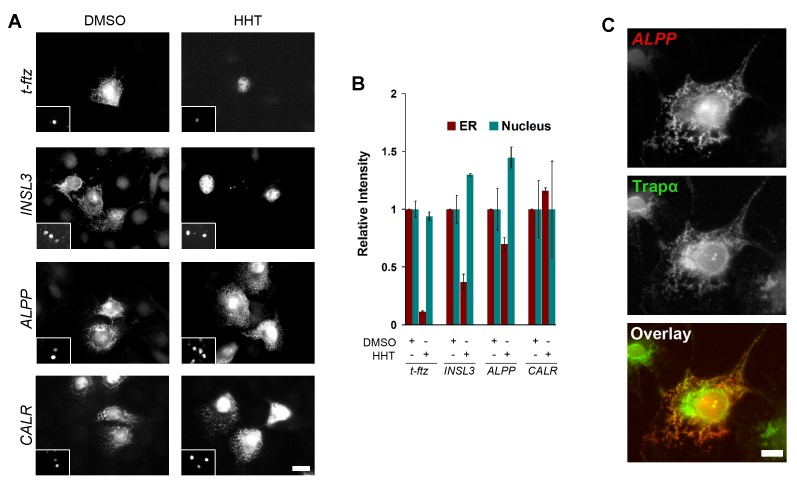
The initial ER-targeting of *ALPP* and *CALR*, but not *t-ftz* or *INSL3*, mRNA occurs independently of translation or ribosomes. (A–B) COS-7 cells were pretreated with DMSO (“Control”) or HHT for 15 min, then microinjected with plasmids containing either the *ALPP*, *INSL3*, *t-ftz*, or *CALR* genes and allowed to express mRNA for 2 h in the presence of DMSO or HHT. To label the microinjected cells, Alexa488-conjugated 70 kD dextran was co-injected (see insets in A). The cells were then extracted with digitonin, fixed, stained for mRNA using specific FISH probes, and imaged (A). The fluorescence intensity of mRNA in the ER and nucleus in the micrographs were quantified (B). Each bar represents the average and standard error of three independent experiments, each consisting of the average integrated intensity of 30 cells over background. (C) COS-7 cells were pretreated with HHT for 15 min, then microinjected with plasmids containing the *ALPP* gene. Cells were then incubated for 2 h in the presence of HHT, then extracted with digitonin, fixed, and then co-stained for *ALPP* mRNA by FISH and for Trapα protein by immunofluorescence. Note the extensive co-localization of *ALPP* mRNA (red) and Trapα (green). All scale bars = 20 µm.

Our data indicated that once certain transcripts are targeted to the ER, they are retained on the surface of this organelle independently of ribosomes. It, however, remained unclear whether the initial targeting step could occur independently of translation. To address this question, cells were pretreated with HHT to inhibit translation and then microinjected with plasmid DNA. Two hours later, the distribution of the newly synthesized mRNA, which was never in contact with functional ribosomes, was assessed. Surprisingly, both *ALPP* and *CALR* mRNA targeted to the ER independently of translation (Figure 4A–B). In contrast *INSL3* mRNA only displayed weak translation-independent targeting activity, while *t-ftz* mRNA failed to target to the ER under these conditions (Figure 4A–B). All of the tested transcripts targeted to the ER in the absence of translation inhibitors (i.e., DMSO treatment). To ensure that any changes in fluorescence were not due to changes in mRNA expression or variability in FISH staining, we monitored the nuclear mRNA levels of each construct, and these did not drastically change between experiments (Figure 4B). The targeting of *ALPP* mRNA to the ER in HHT-treated cells was confirmed by co-localization of the digitonin-resistant transcripts with Trapα (Figure 4C).

From these results, we conclude that certain mRNA species, but not others, are efficiently targeted to the ER independently of translation or ribosome-association. This targeting is also likely to be independent of factors that recognize nascent polypeptides, such as SRP, or ER-resident membrane proteins that bind to ribosomes, such as the Sec61 complex, Sec62/Sec63 complex, and ERj1.

### Identification of Putative mRNA Receptors on the ER

**Figure 5:**
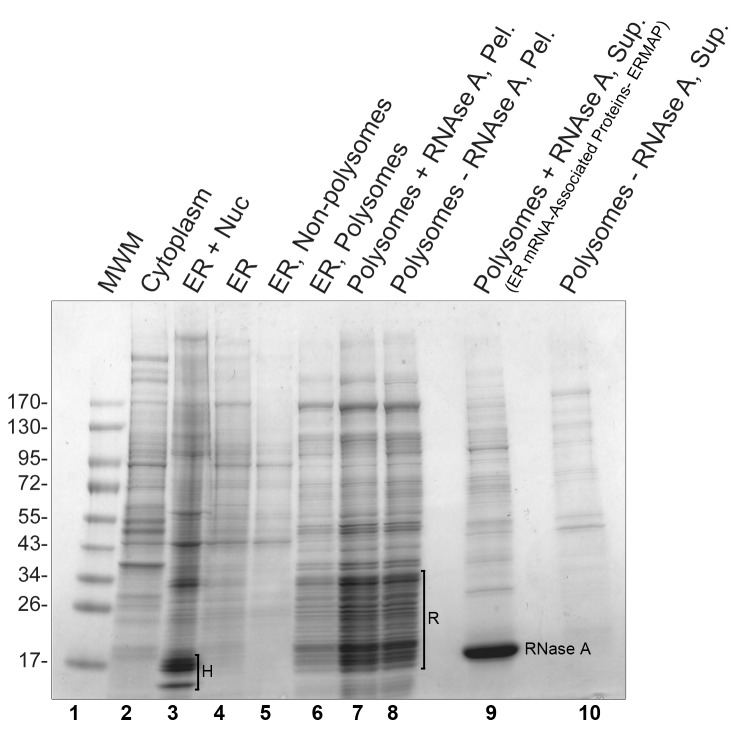
Identification of proteins that associate with ER-derived mRNAs. Cycloheximide-treated U2OS cells were digitonin extracted and centrifuged at low speed to separate cytoplasm (supernatant, lane 2) from ER and nuclear components (pellet, lane 3). The pellet was then extracted with Triton-X100 and centrifuged at low speed to separate ER (supernatant, lane 4) from the nucleus (pellet). Note that the ER fraction is relatively free of histones (“H”), which are found in the ER+Nuc fraction (lane 2). The solubilized ER fraction was then subjected to high-speed centrifugation through a sucrose cushion to separate polysomes (pellet, lane 6) from the rest of the ER (supernatant, lane 5). The polysomes were then treated with either RNase A (lanes 7, 9) or control buffer (lanes 8, 10) at 37°C for 15 min to digest all mRNA. The samples were then subjected to another high-speed centrifugation step to separate ribosomes and associated proteins (pellet, lanes 7–8) from proteins released by RNase A (supernatant, lane 9) or control treatments (supernatant, lane 10). Note that the treatments did not release ribosomal proteins (“R”), which all remained in the pellets (lanes 7,8). All fractions were separated on a 4%–20% gradient SDS-PAGE and visualized by Coomassie blue staining. To estimate protein sizes, molecular weight markers (MWM) were loaded (lane 1, sizes of each band in kD are indicated on the left). Lanes 9 and 10 were cut and sent for mass spectrometry analysis.

To identify proteins that may mediate the ribosome-independent interaction of mRNAs with the ER, cells were subfractionated to enrich for proteins that interact with ER-associated mRNAs. Since the annotation of the human proteome is more complete than that of the African green monkey (from which COS-7 cells are derived), we performed this experiment in human U2OS cells. Trypsinized cyclohexamide-treated cells were washed, then treated with digitonin, and the resulting lysates were subjected to low speed centrifugation to separate the intact ER-nuclear fractions from the rest of the cytoplasm (see the Coomassie stained gel in Figure 5, lanes 2 and 3). This ER-nuclear fraction was then solubilized with TritonX-100 and subjected to low speed centrifugation to remove nuclei (pellet) and generate an ER fraction (supernatant; lane 4). Note that this “ER fraction” is free of histone proteins, which serve as a marker of nuclei (labeled “H,” lane 3). The ER fraction was then subjected to high speed centrifugation through a high percentage sucrose cushion to isolate polysomes (pellet; lane 6), which was then treated with RNase A to digest mRNA and release any associated RNA-binding proteins, including the putative mRNA receptor. The ribosomes, which are mostly resistant to RNase A treatments, and ribosome-interacting proteins, such as the Sec61 complex, were removed from this fraction by high speed sedimentation (pellet; lane 7). Note that this fraction contains all of the ribosomal proteins, which are generally <40 kD (labeled “R”). The remaining supernatant, which consists of proteins that associate with polysomes only when intact mRNA is present (ER mRNA-associated proteins; ERMAP, lane 9), was analyzed by mass spectrometry. As a control we also performed mass spectrometry on proteins that were released after treating polysomes with a buffer that lacked RNase A (lane 10). The experiment was repeated three times, and a list of proteins that were significantly enriched in the ERMAP fraction (*p*<0.05) was compiled (Table 1, for proteins where *p*>0.05, see Table S1). The final list contained 37 different proteins, of which six contained at least one transmembrane segment (p180, kinectin, CLIMP63, transmembrane protein 214, mannosyl-oligosaccharide glucosidase, and magnesium transporter protein 1), 16 were known to bind to RNA, and five function as tRNA synthetases (Table 1). Analyzing our results further we realized that all 10 of the tRNA synthetases that are known to form the large Multisynthetase Complex (MSC) [40] were enriched in the ERMAP (see Table 1). This complex also contains three core components, one of which, AIMP1, was also present in this fraction. Significantly, none of the other 10 tRNA synthetases were enriched in the ERMAP. The ERMAP fraction was also free of any translocon components, suggesting that our preparation was relatively depleted of proteins that directly contact ribosomes. It is also worth noting that the composition of the ERMAP was different from other preparations, such as the ribosome-associated membrane protein (RAMP) fraction [15].

**Table 1:** Proteins enriched in ERMAP fraction.

		RNase	+	RNase	−	
Proteins	Gene ID	AVG	STD	AVG	STD	*p* Value
**Membrane Proteins**						
p180	6238	53.7	5.0	0.7	0.6	0.0001
CLIMP63	10970	33.0	2.6	11.7	2.9	0.0007
Magnesium transporter protein 1	93380	3.3	1.2	0.0	0.0	0.0075
Transmembrane protein 214	54867	8.3	3.1	0.0	0.0	0.0091
Kinectin	3895	79.3	13.4	29.7	13.3	0.010
Mannosyl-oligosaccharide glucosidase	7841	7.3	3.1	1.0	1.0	0.027
**RNA Binding Proteins**						
G3BP2	9908	5.7	0.6	0.0	0.0	0.0001
G3BP1	10146	11.0	1.0	0.7	0.6	0.0001
PRKRA/eIF2alpha protein kinase	8575	6.7	0.6	0.3	0.6	0.0002
PTB-associated-splicing factor	6421	7.0	1.0	0.3	0.6	0.0006
ASF/SF2 SR Protein	6426	5.0	1.0	0.0	0.0	0.0010
Staufen 1	6780	17.3	3.5	1.0	1.0	0.0015
pa2g4^a^	5036	27.0	6.0	1.3	2.3	0.0023
Caprin1	4076	15.7	3.8	0.7	0.6	0.0025
Numatrin/Nucleophosmin^a^	4869	10.0	2.6	0.3	0.6	0.0035
PKR/eIF2alpha protein kinase 2	5610	7.7	2.5	0.0	0.0	0.0062
Tudor/SND1	27044	16.3	0.6	7.7	3.5	0.014
YTH domain protein 1	54915	5.3	2.1	0.3	0.6	0.016
Gemin 5^a^	25929	7.0	2.6	1.0	1.0	0.021
Insulin-like growth factor 2 mRNA bp2	10644	27.0	5.2	16.3	2.1	0.030
RBM4B RNA binding motif protein 4B	83759	4.7	0.6	1.7	1.5	0.034
SYNCRIP, synaptotagmin RNA binding protein	10492	26.3	2.1	17.0	5.3	0.047
**Multisynthetase Complex (MSC)**						
Phenylalanyl-tRNA synthetase beta chain	10056	11.3	3.5	0.3	0.6	0.0059
Isoleucyl-tRNA synthetase	55699	12.7	5.1	1.0	1.0	0.018
Bifunctional (glutamyl, prolyl) tRNA synthetase	2058	18.7	8.5	1.0	1.7	0.024
Phenylalanyl-tRNA synthetase alpha chain	2193	6.7	3.2	0.3	0.6	0.028
Lysyl-tRNA synthetase	3735	7.0	3.6	0.3	0.6	0.034
Arginyl-tRNA synthetase^c^	5917	5.0	4.4	0.0	0.0	0.12
p43, AIMP1^c^	9255	3.0	2.6	0.0	0.0	0.12
Glutaminyl-tRNA synthetase^c^	5859	6.7	6.0	0.0	0.0	0.13
Aspartyl-tRNA synthetase^c^	1615	6.0	5.6	0.0	0.0	0.14
Leucyl-tRNA synthetase^c^	51520	9.0	7.9	1.3	1.5	0.18
Methionyl-tRNA synthetase^c^	4141	8.7	10.0	1.7	2.9	0.31
Total MSC complex	N/A	94.7	45.6	6.0	7.8	0.030
**Others**						
FKBP-25, mTOR/rapamycin binding protein	2287	7.3	1.5	0.3	0.6	0.0018
2′-5′-oligoadenylate synthetase 3	4940	6.3	1.5	0.0	0.0	0.0020
KU70^b^	2547	21.3	1.5	9.0	2.6	0.0022
KU86^b^	7520	19.7	1.5	8.3	2.5	0.0026
Glutamate dehydrogenase 1	2746	15.3	6.7	0.0	0.0	0.016
c1-tetrahydrofolate synthase	25902	12.0	5.3	1.0	1.0	0.024
eIF2-alpha	83939	11.3	2.5	3.3	3.1	0.025
Treacher Collins Syndrome protein	6949	11.7	6.8	0.0	0.0	0.041
eIF2-beta	8890	6.0	1.7	2.0	1.7	0.047
eEF1-alpha1	1915	8.7	3.1	3.7	0.6	0.049

List of significantly enriched proteins (*p*<0.05) in the ERMAP fraction (“RNase+”, see Figure 5, lane 9) compared to the control sample (“RNase−”, see Figure 5, lane 10) as analyzed by mass spectrometry (for a larger list, see Table S1). Included in the table are the Entrez Gene ID, average number (“AVG”), and standard deviation (“STD”) of peptides from the analyses performed on three independent experiments. In addition the average number of peptides from all the components of the MSC was also tabulated, some of which *p*>0.05. The *p* values were determined using a paired two-tailed Student *t* test. On average, 2,427±311 total peptides were recovered from the RNase+ samples, and 1,684±266 total peptides were recovered from the RNase− samples.

a rRNA or snRNA binding protein.

b Primarily involved in DNA binding but has been reported to bind to RNA.

c Members of the MSC where *p*>0.05.

### Over-Expression of p180 Enhances the Ribosome-Independent Association of *t-ftz* mRNA with the ER

The ER can be subdivided into morphologically distinct domains, such as the nuclear envelope, perinuclear sheets, and peripheral tubules [41]. Intriguingly, three of the membrane-bound proteins from the ERMAP fraction—p180, kinectin, and CLIMP63—are abundant proteins that localize to the perinuclear sheet portion of the ER, which is also enriched in translocon components [42] and ribosomes [41],[43]. Interestingly, these three proteins diffuse into the ER-tubules and nuclear envelope after puromycin or pactamycin treatment, indicating that their enrichment in sheets was dependent on the integrity of polysomes and suggesting that they may interact either with ribosomes or mRNA [42]. In particular, p180 seems to be a suitable mRNA receptor candidate. It has a very short luminal N-terminal followed by a single transmembrane domain and a large C-terminal cytoplasmic region that is comprised of two basic domains (a lysine-rich region followed by 54 tandem repeats of a basic decapeptide sequence) and ends in a long coil-coil domain. The highly charged domains are of particular interest as they could potentially bind to the negatively charged phosphate backbone of RNAs. While p180 was initially identified as a ribosome receptor [44], more definitive experiments have shown that the Sec61 translocon complex [15],[16] and not p180 [45],[46] is responsible for the majority of ribosome binding activity present in ER-derived microsomes.

**Figure 6:**
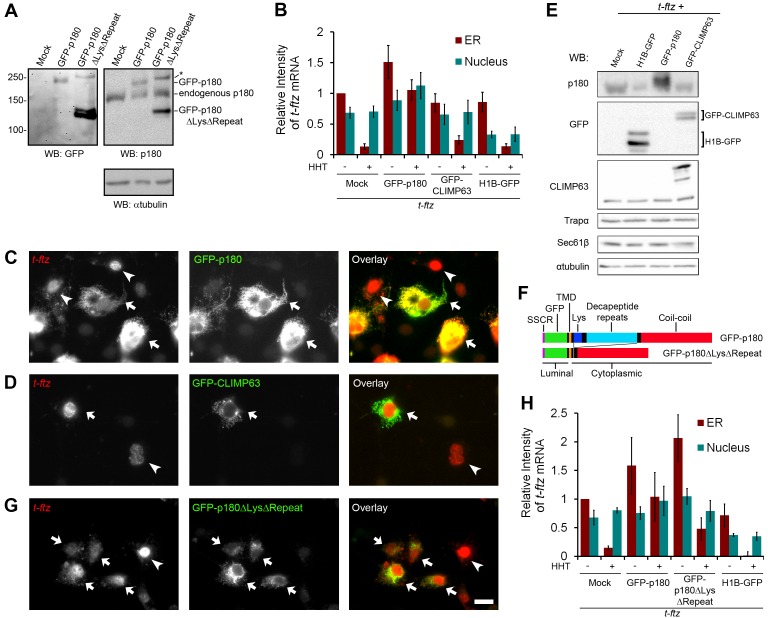
Over-expression of p180 can enhance the ribosome-independent association of *t-ftz* mRNA with the ER. (A) COS-7 cells were transfected with plasmids containing vector alone (“mock”), GFP-p180, or GFP-p180ΔLysΔRepeat. After 18–24 h cell lysates were collected, separated by SDS-PAGE, and immunoblotted for GFP, p180, or αtubulin. The position of molecular weight markers are indicated on the left and proteins are labeled on the right. Note that a high molecular weight band (denoted by an asterisk), which is positive for p180 and GFP, is detected in cells expressing GFP-p180ΔLysΔRepeat. We suspect that this is an aggregate of the over-expressed protein. (B–E, G–H) COS-7 cells were transfected with plasmids containing *t-ftz* gene alone (“mock”), or with various GFP-tagged genes as indicated. The cells were allowed to express *t-ftz* mRNA and GFP-tagged proteins for 18–24 h. Cells were then treated with either control media or HHT for 30 min to disrupt ribosomes, and then extracted, fixed, and stained for *t-ftz* mRNA using specific FISH probes. (B, H) The fluorescence intensity of mRNA in the ER and nucleus in the micrographs were quantified. Each bar represents the average and standard error of three independent experiments, each consisting of the average integrated intensity of 30 cells over background. (C–D, G) Each row represents a single field of HHT-treated cells (30 min) that was imaged for *t-ftz* mRNA and GFP. Cells co-expressing *t-ftz* mRNA and the GFP-tagged protein are denoted by arrows, while cells that expressed only *t-ftz* are indicated by arrowheads. Scale bar = 20 µm. Note that *t-ftz* mRNA remains associated to the ER in cells over-expressing GFP-p180 (C, arrows), but not GFP-CLIMP63 (D, arrows) or in cells expressing *t-ftz* alone (C–D and G, arrowheads). Cells over-expressing GFP-p180ΔLysΔRepeat (G, arrows) show an intermediate phenotype. (E) COS-7 cells that were transfected with plasmids containing *t-ftz* gene alone (“mock”) or with various GFP-tagged genes were lysed, separated by SDS-PAGE, and immunoblotted for p180, GFP, CLIMP63, translocon components (Sec61β and Trapα), or αtubulin. (F) Domain architecture of the GFP-tagged p180 constructs. Both contain the *CALR* SSCR to mediate proper protein translocation (purple), GFP (green), the p180 luminal region which is 7 amino acids long, and the p180 single pass transmembrane domain (TMD, orange). The lysine-rich region (“Lys,” dark blue) and decapeptide repeat region (light blue) are present only in the GFP-p180 construct. Both end with the p180 coil-coil domain (red).

If p180 acts as a non-specific mRNA receptor, one would expect that the over-expression of this protein would enhance the ribosome-independent ER-association of transcripts that normally do not have this activity. With this in mind we monitored the ER-association of *t-ftz* mRNA in COS-7 cells that over-expressed either green fluorescent protein (GFP)-tagged p180 (see Figure 6A) in the presence and absence of HHT. As a control, we monitored the distribution of *t-ftz* mRNA in cells expressing GFP-CLIMP63 and histone 1B-GFP (H1B-GFP). The distribution of H1B-GFP, which binds to DNA in the nucleus, is not affected by extraction and allowed us to identify co-expressing cells after digitonin-treatment. We observed that GFP-p180 over-expression promoted the ER-association of *t-ftz* mRNA in both control and HHT-treated cells (Figure 6B–C). In contrast, over-expression of either GFP-CLIMP63 or H1B-GFP had no effect (Figure 6B,D). Since the expression of GFP-p180 did not significantly affect the cytoplasmic/nuclear distribution (Figure S4A) or the total level (Figure S4B) of *t-ftz* mRNA in intact cells, we could rule out the possibility that the elevated level of ER-bound *t-ftz* was caused by an upregulation of its nuclear export, production, or stability. Moreover, the level of nuclear *t-ftz* FISH signal did not significantly change, except for cells expressing H1B-GFP, and this was likely due to the fact that these cells had a lower overall expression of *t-ftz* mRNA (Figure S4B).

Next, we investigated whether kinectin could act as a general mRNA receptor. In the majority of cells, over-expression of GFP-kinectin did not promote a dramatic increase in the level of ER-bound *t-ftz* mRNA (Figure S5A–B). We, however, did observe a drop in nuclear *t-ftz* mRNA compared to mock co-transfected cells. This was caused by a decrease in the total level of *t-ftz* mRNA (Figure S5C) and not changes in cytoplasmic/nuclear distribution (Figure S5D). As the absolute level of ER-associated *t-ftz* mRNA after HHT-treatment did not change (Figure S5B), despite the drop in its expression level (Figure S5C), we re-evaluated our data. Upon closer inspection we found that in certain cells with high levels of GFP-kinectin, there was an increase in the ribosome-independent ER-association of *t-ftz* (for example, see Figure S5E). Indeed, in HHT-treated cells the level of ER-associated *t-ftz* correlated with the amount of GFP-kinectin, but not H1B-GFP (Figure S5F).

We then examined whether over-expression of p180 affected the ER-association of bulk mRNA. Indeed in cells expressing GFP-p180 there was almost a doubling in the amount of ER-associated mRNA as compared to either H1B-GFP expressing, or untransfected, cells (Figure S6). This was true for both untreated and HHT-treated cells. Although it is likely that a substantial fraction of this enhanced ER-targeting was due to the recruitment of endogenous transcripts, part of the observed increase was probably due to ER-bound *GFP-p180* mRNA, which is not present in the control transfected cells.

Since the expression of p180 has been shown to be important in upregulating secretion in specialized secretory cells [47],[48], our results could have been ascribed to an increase in ribosome-anchoring proteins. However, cells over-expressing GFP-p180 and *t-ftz* did not have altered levels of translocon components, such as Sec61β or Trapα, as seen by immunoblot (Figure 6E). Over-expression of kinectin also had no effect on the levels of Sec61β or Trapα (Figure S5A).

In order to determine whether the lysine-rich region and basic repeats were required for ER-anchoring of mRNA, we over-expressed a version of GFP-p180 that lacks both these domains (GFP-p180ΔLysΔRepeat; Figure 6F) and monitored *t-ftz* distribution. Cells that over-expressed this construct retained about half as much *t-ftz* on the ER after HHT treatment as compared to cells over-expressing p180 (Figure 6F–G, see Figure 6A to compare the expression levels of the two constructs). Notably this level of residual ER-associated *t-ftz* was above control HHT-treated cells, indicating that GFP-p180-ΔLysΔRepeat still had some activity. Interestingly, in the absence of translation inhibitors, cells expressing this construct had elevated levels of ER-associated *t-ftz* mRNA (Figure 6H). This increase was not due to changes in either the nuclear/cytoplasmic distribution or total levels of *t-ftz* mRNA in cells expressing GFP-p180-ΔLysΔRepeat (Figure S4). Thus, it is likely that p180 has the ability to enhance the translation-dependent association of *t-ftz* mRNA with the ER and that this activity does not require the lysine-rich region or the basic repeats.

From these results we conclude that the over-expression of p180 promotes the ribosome- and translation-independent association of mRNAs with the ER. Moreover, our data suggest that this activity is mediated in part by the basic domains found in the cytoplasmic region of p180. In addition, our results indicate that p180 stimulates the recruitment of mRNAs to the ER even in the presence of translating ribosomes, however the basic regions are dispensable for this second activity. In addition, it is likely that kinectin may have some weak ability to anchor mRNAs to the ER independently of ribosomes.

### The Lysine-Rich Region of p180 Associates Directly with RNA In Vitro

**Figure 7:**
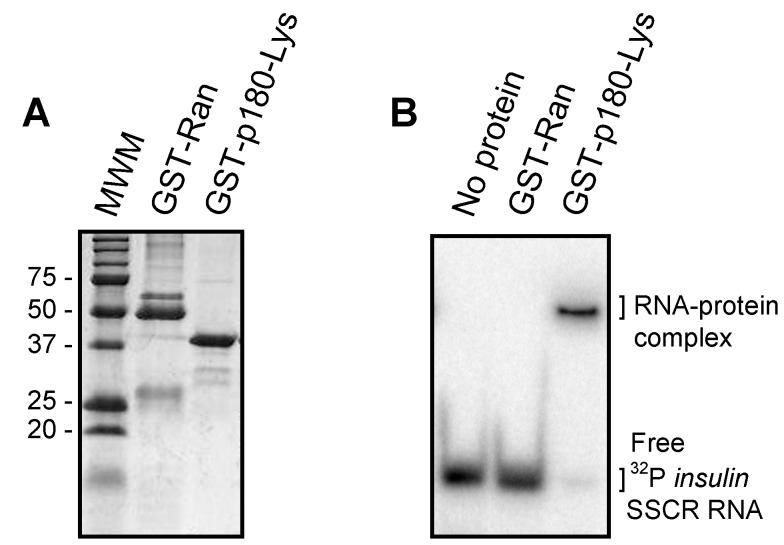
The lysine-rich region of p180 directly associates with RNA in vitro. (A) GST-Ran and GST-p180-Lys were expressed in bacteria, purified using glutathione sepharose, resolved by SDS-PAGE on a 12% acrylamide gel, and stained with Coomassie blue. The size of relevant molecular weight markers (MWM) are indicated on the left. (B) ^32^P-labeled *insulin* SSCR RNA was incubated alone or with either GST-Ran or GST-p180-Lys for 15 min at room temperature and then separated on a 10% non-denaturing TBE gel. Radiolabeled RNA was visualized on a phosphorimager.

Next we investigated whether the basic cytoplasmic domains of p180 could associate directly with RNA in vitro. In support of this idea we found that a bacterially expressed p180 lysine-rich region, fused to glutathione s-transferase (GST-p180-Lys; Figure 7A), could form a complex with a ^32^P-labeled RNA derived from the human *insulin* SSCR (Figure 7B). In contrast, no complex was formed between this RNA and a control protein, GST-Ran (Figure 7A–B). By varying the amount of protein in our binding assay, we estimate that the GST-p180-Lys binds to RNA with an affinity of about 0.8 µM. Since this protein could form complexes equally well with other RNAs, such as a fragment of the human β-globin transcript (unpublished data), it is unlikely that this domain has specificity for any particular sequence. We also tested a peptide containing three copies of the consensus p180 decapeptide repeats; however, we did not observe any complex between this reagent and any of the tested RNAs (unpublished data). This result suggests that the repeats are not critical for mRNA interaction, although we could not rule out the possibility that the peptide, which is 30 amino acids in length and predicted to be disordered, failed to adopt some particular confirmation that is required for RNA-interaction.

### p180 Is Required for the Efficient Association of mRNA to the ER

**Figure 8:**
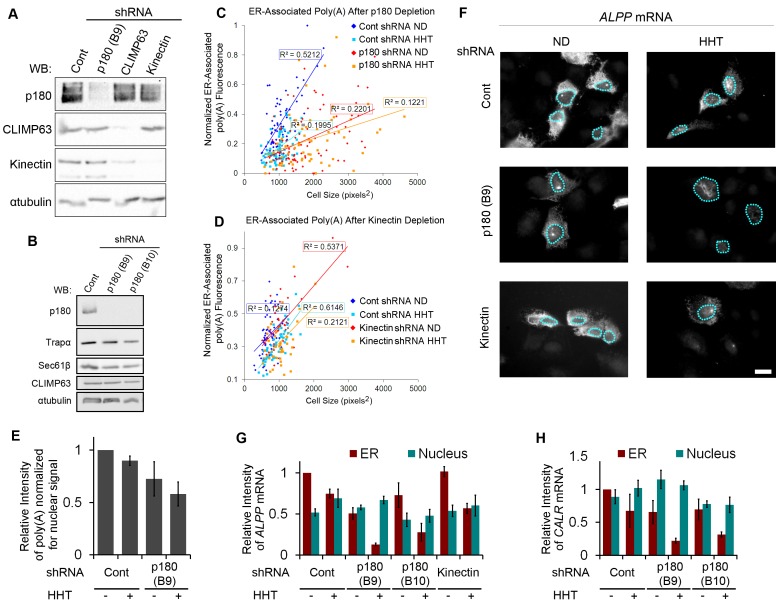
p180 is required for the ER-association of mRNA. (A–H) U2OS cells were infected with specific shRNAs against p180 (shRNA clones B9 and B10), kinectin or CLIMP63, or with control lentivirus (“Cont”). (A–B) Cell lysates were separated by SDS-PAGE and immunoblotted for p180, CLIMP63, kinectin, αtubulin, Trapα, and Sec61β. (C–E) Cells depleted of p180 (Clone B9; C, E) or kinectin (D), or infected with control lentivirus (“cont’; C–E), were treated with control media (no drug, “ND”) or HHT for 30 min, then extracted with digitonin and stained for poly(A) mRNA using poly(dT) FISH probes. (C–D) For each cell the total level of ER-associated poly (A) FISH signal (normalized from the background (0), to the brightest cell in the entire experiment (1); *y*-axis) was plotted against cell size (pixels squared, *x*-axis). For each data set a regression line was plotted and the coefficient of determination (R^2^) was indicated. (E) The ratio of ER to nuclear poly(A) fluorescence was quantified and normalized. Each bar represents the average and standard error of five independent experiments, each consisting of the average of >30 cells. (F–H) Cells were depleted of p180 or kinectin with specific shRNAs, or infected with control lentivirus (“Cont”), then transfected with plasmids containing either the *ALPP* (F–G) or *CALR* (H) gene. The cells were allowed to express mRNA for 18–24 h, then treated with control media (no drug, “ND”) or HHT for 30 min, and then extracted with digitonin. Cells were then fixed, stained for mRNA using specific FISH probes against the exogenous mRNA, and imaged. Nuclei are outlined with blue dotted lines. Scale bar = 20 µm. (G–H) The fluorescence intensity on the ER and nucleus were quantified. Each bar represents the average and standard error of three independent experiments, each consisting of the average integrated intensity of 30 cells over background.

Next, we depleted p180, kinectin, or CLIMP63, by infecting U2OS cells with lentiviruses that deliver short hairpin RNAs (shRNAs) that are processed into small interfering RNAs directed against the human genes of interest. These treatments effectively depleted p180 and kinectin (Figure 8A), but the level of CLIMP63 after shRNA knockdown was quite variable. In addition depletion of CLIMP63 occasionally resulted in a decrease in kinectin levels (Figure 8A), however this was not consistent throughout all our experiments. Note that in these preliminary experiments p180 was depleted with shRNA clone B9.

Previously it was shown that the depletion of these three factors had no obvious effect on ER-morphology except that CLIMP63 depletion decreased the average width of the ER lumen [42]. Moreover, p180 depletion did not significantly affect the level of translocon components, such as Sec61β or Trapα (Figure 8B). We did observe, however, that the average cell size increased after p180 depletion (Figure S7A). As a consequence, the total area occupied by the ER and the nucleus also increased (Figure S7B). Since we also observed an increase in bi-nucleate cells (unpublished data), it is possible that p180 is required to complete cytokinesis, which would explain the increase in cell and nuclear sizes. We next determined whether p180 was required for the ER-association of bulk mRNA to the ER using poly(dT) FISH probes. To control for changes in cell size, we imaged and quantified poly(A) FISH staining in extracted cells and plotted the total fluorescence intensity in the ER versus the cell area for each cell. When cells of a similar size were compared, we observed a decrease in the steady-state levels of ER-associated mRNA after p180 depletion (Figure 8C). In contrast, kinectin depletion had no effect on the level of ER-associated mRNA (Figure 8D). When p180 knockdown cells, which already had a low level of ER-associated mRNA, were treated with HHT the amount of mRNA on the ER only decreased slightly (Figure 8C). In contrast when control or kinectin-depleted cells were treated with HHT, the amount of ER-associated mRNA dropped but was still higher than what was seen in p180 knockdown cells with or without HHT treatment (Figure 8C–D). In order to quantify the amount of ER-associated mRNA while controlling for changes in cell size and variation in FISH signals between experiments, we normalized the integrated fluorescence intensities of FISH signal in the ER to the nucleus for each cell. We found that p180-depleted cells had significantly less ER-associated mRNA in comparison to control cells (Figure 8E). Using this analysis, we found that HHT treatment reduced the amount of ER-associated mRNA in both control and p180 knockdown cells, however even in the absence of p180 and translation, there was still ER-associated transcripts (Figure 8E).

From these experiments we conclude that p180 promotes the efficient anchoring of bulk mRNA to the ER, however as p180 depletion did not abolish the ribosome-independent ER-association of mRNA, it is likely that other mRNA receptors exist.

### p180 Is Required for the Translation- and Ribosome-Independent Maintenance of *ALPP* and *CALR* mRNA at the ER

We then tested the requirement of p180 for ER-association of specific transcripts by analyzing the level of mRNA in the cytoplasm and the nucleus by FISH. p180 depletion reduced the association of *ALPP* to the ER in both control and HHT-treated cells (Figure 8F–G). In contrast to poly(A) staining, we did not observe an increase in nuclear *ALPP* in the knockdown cells. We believe that this is due to the fact that the total amount of *ALPP* mRNA produced per transfected cell did not change despite the increase in cell and nuclear size. Depletion of p180 with a second shRNA construct (clone B10, Figure 8B) also reduced the association of *ALPP* to the ER in both control and HHT-treated cells (Figure 8G). In contrast, depletion of kinectin had no effect on the level of ER-associated *ALPP* mRNA in either control or HHT-treated cells (Figure 8F–G). p180 depletion by either shRNA clone also reduced the ER-association of *CALR* mRNA in both control and HHT-treated cells (Figure 8H).

From these experiments we concluded that the ribosome-independent anchoring of *ALPP* and *CALR* mRNA to the ER requires p180.

## Discussion

The work presented in this article provides, to our knowledge, the first molecular insight into how a large fraction of ER-anchored transcripts are maintained on the surface of this organelle independently of ribosomes in mammalian cells. Importantly, we demonstrate that the degree of ribosome- and translation-independent targeting and maintenance at the ER varies greatly between different transcripts. We then provide evidence that p180 acts as a general mRNA receptor on the ER. Over-expression of a GFP-tagged version of this protein potentiates mRNA-ER interaction, while its depletion reduces the amount of ER-associated mRNA. Finally we demonstrate that p180 is required for the ribosome-independent anchoring of *ALPP* and *CALR* mRNAs. Although p180 appears to be a metazoan-specific gene, recent findings have suggested that mRNA may be anchored directly to membranes in prokaryotes [49], suggesting that the ribosome-independent association of mRNAs to membrane-bound receptors is universally conserved [50]. Indeed our data suggest that other mRNA receptors for the ER exist in mammalian cells. One potential candidate that we have yet to rule out is kinectin. Although its depletion has little to no effect on the distribution of bulk poly(A) or *ALPP* mRNA (Figure 8D,F,G), its over-expression promoted a small but detectable increase in the ribosome-independent association of *t-ftz* mRNA with the ER (Figure S5). Moreover, kinectin has a cytoplasmic lysine-rich domain that resembles the RNA-binding region of p180. Experiments are currently underway to determine the exact contribution of kinectin to this process.

Our results indicate that p180- and ribosome/translation-dependent targeting mechanisms act synergistically to enhance ER-anchoring of mRNAs (Figures 6B,H and 8E,G–H). In agreement with our results, several groups have demonstrated that p180 expression promotes secretion [47],[48]. Interestingly, the over-expression of p180 in budding yeast, which does not express any endogenous p180-like proteins, leads to the proliferation of ER, the enhancement of mRNA-ER association, and an increase in the half-life of ER-bound transcripts [51],[52]. Furthermore, while ER-proliferation is stimulated by the over-expression of a version of p180 that lacks the basic domains, the enhanced mRNA-ER association requires these domains [52]. Although these results have been ascribed to the ability of p180 to directly recruit ribosomes, our data support an alternative model where the basic domains of p180 associate directly to mRNA, thus enhancing the partitioning polysomes to the ER. It is also likely that p180 may have other domains that mediate mRNA-ER association in mammalian cells. Indeed we found that the expression of p180 lacking any basic regions (GFP-p180-ΔLysΔRepeat) can promote ribosome-dependent ER-anchoring of mRNA (Figure 6G–H). Taken together, our data suggest that the coil-coil domain may function primarily within the context of translation to enhance ER-association. This result is in agreement with a recent study performed in collagen secreting cells which demonstrated that p180 can promote the assembly of ER-bound polysomes, but that this activity did not require its basic domains [53].

Importantly we demonstrate that p180 has a lysine-rich region that can directly bind to RNA in vitro (Figure 7), likely through non-specific interactions with the mRNA backbone. In light of this we predict that p180 acts in concert with proteins that recognize specific RNA sequences to recruit particular mRNAs, such as *ALPP* and *CALR*, to the ER. Many candidate proteins that could fulfill this function are likely found in the ERMAP fraction (Table 1). Further studies will be required in order to determine whether these other ERMAP proteins play a role in mediating specific interactions between mRNAs and the ER.

Intriguingly, our analysis also uncovered that the MSC, containing 10 tRNA synthetases, and eEF1A1, which delivers charged tRNA to the ribosome, co-fractionate with ER-associated mRNAs (Table 1). Recently it has been shown that the MSC not only co-fractionates with polysomes in a sucrose gradient, but also is distributed in a reticular pattern that is resistant to cellular extraction with digitonin [54], suggesting that this complex associates predominantly with ER-bound mRNA. It is possible that the MSC may mediate the direct delivery of charged tRNAs to the ribosome (known as “tRNA channeling” [55]), and thus be responsible for the enhanced rate of protein synthesis experienced by ER-targeted transcripts [56].

Finally, it is likely that mRNA receptors may restrict various transcripts to particular subdomains of the ER. As mentioned previously, many asymmetrically localized mRNAs are anchored by mRNA receptors that are present in particular ER-subdomains. This is best illustrated in rice endosperm cells, where the transport and anchoring of specialized mRNAs to specific ER-domains is dependent on an RNA binding protein that is homologous to SND1/Tudor [4],[57], a protein we identified in the ERMAP fraction (Table 1). Interestingly, the differential distribution of ER-bound transcripts is also seen in mammalian cells. For example, *t-ftz*, but not *ALPP*, appears to be excluded from the nuclear envelope (X. Cui and A. Palazzo, unpublished observations). Moreover unlike translocon-associated proteins, which are concentrated in ER-sheets [41],[42], poly(A) appears to be distributed more evenly across all of the ER (for example, compare the distribution of Trapα and poly(A) in Figure 1A), suggesting that the association of certain mRNAs with ER-tubules is mediated by interactions with some additional unidentified RNA receptor(s). Ultimately, the restricted localization of certain mRNAs may help to target newly synthesized proteins to distinct areas of the ER. This may be critical for the proper localization of proteins with polarized distributions [2]–[5], especially for secretory proteins that are exported at specific ER exit sites and are processed in specialized Golgi outposts [58], which are present at peripheral cellular sites, such as in neuronal dendrites. The restricted distribution of particular ER-bound transcripts may also be important to confine certain newly synthesized ER-resident proteins which function in certain subdomains of this organelle, such as the nuclear envelope [59]. Again further analysis of RNA-binding proteins (particularly those found in the ERMAP fraction), and their interacting RNA elements, will be required for a clearer understanding of these processes.

## Materials and Methods

### DNA Plasmids, Construction of Gene Fusions

Full-length human *INSL3* (GeneID: 3640), *CALR* (GeneID: 811), and *ALPP* (GeneID: 250) cDNAs (i.e., including both the open reading frames and complete untranslated regions), inserted into the pSPORT6 vector, were purchased from Open Biosystems. *cyto-ALPP* was constructed by amplifying nucleotides 123–1585 of the *ALPP* cDNA by PCR. The PCR product was then inserted between the frame-shifted MHC SSCR and the *ftz* ORF in the *fs-ftz* pCDNA3 construct [30] using restriction-free PCR subcloning [60]. GFP-p180, GFP-CLIMP63, and H1B-GFP were described previously [42],[61]. The GFP-p180ΔLysΔRepeat construct, which lacks nucleotides 175–2028 of the p180 ORF, was constructed from GFP-p180 using restriction-free PCR-based deletion [60].

### Cell Culture, Extraction, FISH, Indirect Immunofluorescence, and Imaging

Cell culture, DNA transfection, and DNA/mRNA microinjection were performed as previously described [30],[37],[62]. All reagents were purchased from Sigma Aldrich unless specified. HHT was used at 5 µM, puromycin was used at 200 µM, and pactamycin was used at 200 nM for the indicated times. For extractions, cells were rapidly washed twice in 37°C CHO buffer (115 mM Potassium Acetate, 25 mM HEPES pH 7.4, 2.5 mM MgCl_2_, 2 mM EGTA, 150 mM Sucrose), then incubated in CHO+0.025% digitonin (±20 mM EDTA) at 37–42°C for 10 s. Extraction was terminated by the addition of excess PBS+4% paraformaldehyde at 37°C. FISH and immunostaining were performed as previously described [30],[37],[62]. The deoxyoligonucleotides used to stain bulk mRNA (polymer of 60 dT; poly(dT)), *ftz* (GTCGAGCCTGCCTTTGTCATCGTCGTCCTTGTAGTCACAACAGCCGGGACAACACCCCAT), *INSL3* (GGGCCCCCGCACACGCGCACTAGCGCGCGTACGAAGTGGTGGCCGCA), *CALR* (CAGATGTCGGGACCAAACATGATGTTGTATTCTGAGTCTCCGTGCATGTC), and *ALPP* (CAGCTTCTTGGCAGCATCCAGGGCCTCGGCTGCCTTTCGGTTCCAGAAG) were conjugated at the 5′ end with Alexa546 (Integrated DNA Technologies).

To ensure that poly(dT) signal was dependent on mRNA, coverslips with FISH-stained cells were incubated in 1× RNase H reaction buffer (NEB) with (“+”) or without (“−”) RNase H (New England Biolabs) at 10 units per coverslip for 1 h at 37°C.

For immunofluorescence, fixed cells were probed with polyclonal rabbit antibody against Adenosine Kinase (1∶250 dilution, [32]) and FISH-stained samples were probed with the human antibody against the RPLP0 ribosomal protein (“P0,” 1∶50 dilution [63]) or the rabbit polyclonal against Trapα (1∶500 dilution [33]) and then stained with Alexa488- or Alexa647-conjugated secondary antibodies (1∶200 dilution, Invitrogen). Microscopy, imaging, and quantification were performed as previously described [30],[37],[62].

### Lentiviral-Mediated shRNA Knockdown

Plasmids encoding shRNA against p180 (clone B9 - TRCN0000117407, clone B10 - TRCN0000117408, Sigma), CLIMP63 (clone TRCN0000123296), kinectin (clone TRCN0000063520), or empty vector (pLKO.1) were transfected into the HEK293T cells together with the accessory plasmids, VSVG and Δ8.9, to generate Lentivirus carrying specific shRNA plasmids. Lentivirus was harvested from the medium 24 h and 48 h post-transfection by filtering through a 0.44 µm filter. For infection, Lentivirus was applied to U2OS cells with 8 µg/ml hexamethrine bromide. Puromycin was applied to the cell 24 h post-infection at 2 µg/ml to select for infected cells, and puromycin containing medium was changed every other day. Cell lysates were collected 5 d post-infection to assess the level of knockdown, and the cells were used for various experiments as described.

### Cell Fractionation

To isolate fractions, cells were first pre-treated with puromycin (200 µM), cycloheximide (200 µM), or control media (0.1% Dimethyl sulfoxide; DMSO) for 30 min; then trypsinized, pelleted at 800 *g* for 2 min, washed 3 times with ice cold PBS+Soy Bean Trypsin Inhibitor (0.1 mg/ml; Sigma), ±200 µM cyclohexamide, ±200 µM puromycin; washed once with ice cold Phy Buffer (150 mM Potassium Acetate, 5 mM Magnesium Acetate, 20 mM HEPES pH 7.4, 5 mM DTT, and protease inhibitors, with either DMSO, 200 µM puromycin, 200 µM cyclohexamide, and/or 20 mM EDTA); and then resuspended in cold 0.5 ml Phy Buffer again with indicated reagents. Cells were extracted by adding an equal volume (0.5 ml) of cold Phy Buffer+0.2% digitonin. Lyastes were then centrifuged at 800 *g* for 2 min to produce a suspension (cytoplasmic fraction) and pellet (ER+nuclear fraction). The pellet was then washed once with cold Phy Buffer, then resuspended in cold 0.5 ml Phy Buffer and extracted by adding an equal volume (0.5 ml) of Phy Buffer+0.5% TritonX-100. This sample was then centrifuged at 800 *g* for 2 min to produce a suspension (ER fraction) and pellet (nuclear fraction). Both cytoplasmic and ER fractions were then centrifuged at 10,000 *g* for 10 min to remove contaminating organelles such as mitochondria and nuclei. These fractions were either immediately analyzed for protein (Figure 1F) or mRNA (Figures 1G, 3) or further fractionated (Figure 5) by layering the ER fraction over 500 µl of Phy Buffer supplemented with 80% sucrose and cyclohexamide and centrifugation at 100,000 *g* for 60 min to produce a suspension (ER, non-polysomes) and a pellet (ER-derived polysomes). The polysome fraction was then resuspended in 50 µl of Phy Buffer ±0.5 µl RNAse A (1 mg/ml) and incubated at room temperature for 15 min. The polysome samples were then centrifuged for 60 min at 100,000 *g* to produce a suspension (ER mRNA-associated proteins) and a pellet (ribosomes and associated proteins). Cell fractions were mixed with Laemmli sample buffer, heated to 70°C for 5 min, and separated by SDS-PAGE on a 4%–20% gradient gel. For protein identification the gel was Coomassie stained and sequenced by microcapillary liquid chromatography tandem mass spectrometry (Taplin Mass Spectrometry Facility, Harvard Medical School).

### Immunoblotting

Cell lysates from various culture cell lines were collected in RIPA buffer (50 mM Tris-HCL, 150 mM NaCl, 0.1%SDS, 0.5% Triton-X100, 1 mM PMSF, and 1× protease inhibitor cocktail, Roche) from various cell lines. Proteins were separated by SDS-PAGE and transferred onto the nitrocellulose membrane and probed with antibodies specific to p180 (polyclonal, 1∶1,000 dilution, Sigma), CLIMP63 (polyclonal, 1∶1,000 dilution, Sigma), kinectin (polyclonal, 1∶1,000 dilution, Sigma), GFP (polyclonal, 1∶1,000 dilution, Invitogen), S6 (rabbit monoclonal 5G10, 1∶250 dilution, Cell Signalling), Trapα (rabbit polyclonal, 1∶5,000 dilution [33]), Sec61β (rabbit polyclonal, 1∶5,000 dilution [64]), or αtubulin (mouse monoclonal DM1A, 1∶20,000 dilution, Sigma).

### In Vitro Analysis of mRNA Levels

To isolate RNA, cell fractions were treated with five times the volume of TRIzol (Invitrogen) and then centrifuged at 10, 000 *g* for 10 min. To the supernatant, one times the original volume of chloroform was added and then centrifuged at 10, 000 *g* for 10 min. RNA was precipitated from the aqueous phase by adding one volume of isopropanol, one-twentieth volume of ammonium acetate, and 2 µl of 10 mg/ml glycerol as a non-specific carrier, and incubating the mixture at −80°C for 1 h. The sample was centrifuged at 20,000 *g* for 30 min, and the pellet was washed with 50 µl of 70% ethanol. The precipitate was dried and then resuspended in 50 µl of water.

For quantification of total RNA (Figure 1G), 1 µl of the RNA preparation was converted to cDNA using Superscript reverse transcriptase II (Invitrogen) in the presence of α[^32^P]-UTP (Perkin Elmer) and a 40-nucleotide-long dT primer at 42°C using the manufacturer's protocol. After 2 h the reaction was terminated by incubating the samples at 80°C for 15 min. cDNA products were separated from free nucleotides using a G25 column (GE Healthcare). Counts per minute (CPM) were determined by a scintillation counter. Background CPM, as calculated from a cDNA sample prepared from an RNA-free reaction, was subtracted from each tube. When increasing amounts of poly-adenylated mRNAs were added to the reaction, a linearly proportional amount of radioactive cDNA product was recovered (unpublished data).

For quantitative RT-PCR (Figure 3) ER and cytoplasmic fractions were converted to cDNA with Superscript reverse transcriptase II (Invitrogen) and random hexanucleotides at 37°C using the manufacturer's protocol. After 2 h the reaction was terminated by incubating the samples at 80°C for 15 min. Samples were treated with RNAse, and cDNA products were purified by phenol chloroform extraction followed by ethanol precipitation. The libraries were assayed by the Biopolymers Facility at Harvard Medical School using kits from Applied Biosystems for *βactin* (Hs03023943_g1, GeneID: 60), *BiP* (Hs99999174_m1, Gene ID: 3309), *Calreticulin* (Hs00189032_m1), *Fatty Acid Desaturase 3* (Hs00222230_m1, GeneID: 3395), *Integrin-β1* (Hs00236976_m1, GeneID: 3688), *Interleukin 7* (Hs00174202_m1, GeneID: 3574), *Inositol-3-Phosphate Receptor 3* (Hs01573555_m1, GeneID: 3710), *Manosidase 2A1* (Hs00159007_m1, GeneID: 4124), Sec61α (Hs00273698_m1, GeneID: 29927), *Transferrin Receptor* (Hs00174609_m1, GeneID:7037), *Trapα* (Hs00162340_m1, GeneID: 6745), and 28S rRNA (custom design 4331348, GeneID: 100008589). Note that 28S rRNA was used to normalize samples as the large ribosomal subunit remains bound to the ER after puromycin treatment [35].

### Expression and Purification of p180 Fragments and GST Proteins

The p180 lysine-rich domain (amino acid residues 52–136) was amplified from pCDNA-GFP-p180 and inserted into pGEX2T vector (Novagen) downstream of an N-terminal GST-tag to create GST-p180-Lys. GST-protein was expressed in *Escherichia coli* BL21 by growing 500 ml of culture in LB until it reached an OD_600_ of 0.6, then adding 500 ml ice-cold LB (with 4% ethanol and 1 mM IPTG) and incubating the culture at 18°C for 18 h. Cell pellets were resuspended in 20 ml of protein purification buffer (1%(v/v) TritonX-100, 50 mM HEPES pH 8.0, 5 mM MgCl_2_, 100 mM KCl, and 500 mM NaCl, 0.1 mg/ml PMSF) and lysed by French press. The recombinant proteins were purified on glutathione sepharose beads (Sigma) and eluted with 10 mM reduced glutathione dissolved in PBS buffer.

### RNA Synthesis and EMSA

For EMSA experiments, the SSCR sequence of insulin (ACCATGGCCCTGTGGATGCGCCTCCTGCCCCTGCTGGCGCTGCTGGCCCTCTGGGGACCTGACCCAGCCGCAGCC), were cloned by restriction free cloning between the *HinDIII* and *XhoI* sites of pCDNA3. These plasmids were digested with XhoI and transcribed using T7 RNA polymerase (Ambion) in the presence of 0.4 µCi/µl ^32^P-GTP. Synthesized RNA products were denatured in 50% formamide and resolved by polyacrylamide gel electrophoresis (TBE, 10% acrylamide; acrylamid/bisacrylamide ration of 19∶1) and then gel isolated. The labeled RNA was incubated alone, or with an excess of either GST-Ran or GST-p180-Lys (final concentration of the protein 12 µM) in PBS with 10 µg/ml denatured yeast tRNA (Sigma) at room temperature for 20 min. The free and complexed RNAs were separated by native polyacrylamide gel electrophoresis (TBE, 10% acrylamide; acrylamid/bisacrylamide ratio of 19∶1), and labeled RNA was visualized using a Typhoon phosphorimager (GE Healthcare).

## Supporting Information

Figure S1 Visualization of ER-targeted mRNAs and ribosomes. (A) The nuclei of COS-7 cells were microinjected with *t-ftz* mRNA and Alexa488-conjugated 70 kD dextran (see inset) to label the microinjected compartment. The cell was incubated at 37°C for 2 h to allow for the nuclear export and targeting of the mRNA to the surface of the ER. A single cell is shown in (A) co-stained for *ftz* mRNA using specific FISH probes and for the ER marker Trapα by immunofluorescence. Note the extensive co-localization between the mRNA (green) and Trapα (red). Scale bar = 15 µm. (B–E) The nuclei of COS-7 cells were microinjected with either *t-ftz* (B–C) or *c-ftz-i* (D–E) mRNA and Alexa488 conjugated 70 kD dextran to label the microinjected compartment (insets). After incubating the cells at 37°C for 1 h, the cells were either directly fixed (“Non-ext”, B, D) or first extracted with digitonin (“Ext”, C, E) and then fixed. The cells were stained for *ftz* mRNA using specific FISH probes. Scale bar = 15 µm. (F–G) COS-7 cells were either directly fixed (“Non-ext”, F) or first extracted with digitonin (“Ext”, G) and then fixed and stained for Adenosine Kinase (“AdK”). Scale bar = 20 µm. (H–I) COS-7 cells were either directly fixed (“Non-ext”, H) or first extracted with digitonin and then fixed (“Ext”, I) and then fixed and stained for ribosome RPLP0 protein and the ER marker Trapα. Note the extensive co-localization between ribosomes (green) and Trapα (red) after extraction (I). Scale bar = 15 µm.
(TIF)

Figure S2 *CALR* mRNA, but not *t-ftz* mRNA, remains associated with the ER independently of ribosomes and translation. COS-7 cells were transfected with plasmids encoding either the *t-ftz* (A) or *CALR* (B) genes and were allowed to express mRNA for 18–24 h. The cells were then treated with control media (“Cont”), puromycin (“Puro”), or HHT for 30 min, and then extracted with digitonin alone or with 20 mM EDTA. Cells were then fixed, stained for mRNA using specific FISH probes, and imaged. Note that ER, but not nuclear, staining of *t-ftz* mRNA was lost after cells were treated with HHT or puromycin/EDTA (arrows). (C) COS-7 cells were transfected with plasmids encoding the *ALPP* gene and were allowed to express mRNA for 18–24 h. Cells were then treated with HHT for 30 min, then extracted with digitonin, fixed, and stained for *ALPP* by FISH and Trapα by immunofluorescence. Note the extensive co-localization of *ALPP* mRNA (red) and Trapα (green) in the overlay. (C) All scale bars = 20 µm.
(TIF)

Figure S3 *ALPP* mRNA, but not *t-ftz* and *INSL3* mRNAs, remains associated with the ER after cells were treated with pactamycin to disrupt the mRNA-ribosome association. (A) COS-7 cells were treated with control media (“Cont”) or pactamycin (“Pact”) for 15 min and then incubated in ^35^S-methionine to label newly synthesized proteins for an additional 15 min. Cell lysates were collected and separated by SDS-PAGE. Total proteins were visualized by Coomassie blue stain, and newly synthesized proteins were detected by autoradiography. Molecular weight markers are indicated on the left (“MWM”). (B–D) COS-7 cells were transfected with plasmids encoding either the *t-ftz* (B), *INSL3* (C), or *ALPP* (D) genes and were allowed to express mRNA for 12–18 h. The cells were then treated with control media or pactamycin for 30 min, and then either directly fixed (“Non-extracted”) or first extracted with digitonin (“Extracted”) and then fixed. Cells were stained for mRNA using specific FISH probes and imaged. Scale bar = 15 µm.
(TIF)

Figure S4 Nuclear export of *t-ftz* mRNA remains unchanged in co-transfected cells, but total *t-ftz* mRNA levels decrease in cells expressing H1B-GFP. (A–B) COS-7 cells were transfected with either plasmids containing *t-ftz* alone or in combination with plasmids containing GFP-p180, GFP-CLIMP63, or H1B-GFP. Cells were allowed to express for 18–24 h, fixed, and stained for *t-ftz* mRNA using specific FISH probes. Note that the cells were not extracted prior to fixation. (A) The fraction of *t-ftz* mRNA in the cytoplasm and nucleus in co-transfected cells. (B) The total level of *t-ftz* mRNA in the co-transfected cells, normalized to cells expressing *t-ftz* alone. Each bar consists of the average and standard deviation of 30–35 cells.
(TIF)

Figure S5 GFP-kinectin over-expression slightly enhances the ER-association of *t-ftz* mRNA after ribosome dissociation. (A) COS-7 cells were transfected without (“mock”) or with plasmids containing GFP-p180 and then lysed after 18–24 h. Cell lysates were separated by SDS-PAGE and immunoblotted for kinectin, GFP, αtubulin, and translocon components (Sec61β and Trapα). (B–F) COS-7 cells were transfected with plasmids containing *t-ftz* alone (“mock”) or in combination with GFP-kinectin or H1B-GFP. After 18–24 h cells were treated with control medium (B–D) or HHT for 30 min (B,E–F). Next, the cells were either first extracted with digitonin and then fixed to assess ER-association (B, E–F) or directly fixed to assess the total mRNA (C–D). After staining for *t-ftz* mRNA using specific FISH probes, cells were imaged. (B) The fluorescence intensity of *t-ftz* mRNA in the ER and nucleus in extracted cells. (C) The total level of *t-ftz* mRNA in unextracted cells. (D) The fraction of *t-ftz* mRNA in the cytoplasm and nucleus. (B–D) All data points are normalized to “mock” (cells expression *t-ftz* alone). Each bar represents the average and standard error of three independent experiments, each consisting of the average integrated intensity of 30 cells over background. (E) A single field of HHT-treated cells (30 min) that was imaged for *t-ftz* mRNA and GFP-kinectin. Scale bar = 20 µm. Note that *t-ftz* mRNA remains associated to the ER in cells with very high levels of GFP-kinectin (arrow), but not those with low levels (arrowhead). (F) For each HHT-treated cell the total level of ER-associated *t-ftz* FISH signal (normalized from the background (0), to the brightest cell in the experiment (1); *y*-axis) was plotted against total integrated GFP signal (normalized from the background (0), to the brightest cell in the experiment (1); *x*-axis).
(TIF)

Figure S6 GFP-p180 over-expression enhances the ER-association of bulk poly(A) mRNA. COS-7 cells were transfected with either plasmids containing GFP-p180 or H1B-GFP and then fixed after 18–24 h. Cells were then treated with either control medium or HHT for 30 min to disassemble ribosomes, and then extracted, fixed, and stained poly(A) mRNA using poly(dT) FISH probes. (A) A single field of HHT-treated cells that was imaged for poly(A) mRNA and GFP. Cells expressing GFP-p180 are denoted by arrows, while untransfected cells are indicated by arrowheads. A cell with low GFP-p180 expression is denoted by an asterisk. Scale bar = 20 µm. The fluorescence intensity of mRNA in the ER was quantified (B). Each bar represents the average and standard error of three independent experiments, each consisting of the average integrated intensity of 50 cells over background.
(TIF)

Figure S7 Depletion of p180 in U2OS cells increases cell size. The area in square pixels of the whole cell (A) or the ER and the nucleus (B) were measured in U2OS cells depleted of p180 with specific shRNAs or infected with control lentivirus and treated with control media or HHT for 30 min prior to digitonin extraction. All values were normalized to the size of either control cells (A) or ER (B). Each bar represents the average and standard error of four independent experiments, each consisting of the average from >30 cells.
(TIF)

Table S1 Proteins enriched in the ERMAP fraction (*p*>0.05). List of additional proteins (*p*>0.05) identified in the ERMAP fraction in addition to proteins listed in Table 1. Included in the table are the Entrez Gene ID, average number (“AVG”), and standard deviation (“STD”), of peptides from the analyses performed on three independent experiments. The *p* values were determined using a paired two-tailed Student *t* test.
(RTF)

## Abbreviations

AdK: adenosine kinase
ALPP: placental alkaline phosphatase
CALR: calreticulin
DMSO: dimethyl sulfoxide
FISH: fluorescent in situ hybridization
Ftz: fushi tarazu
GFP: green fluorescent protein
GST: glutathione s-transferase
HHT: homoharringtonine
INSL3: insulin-like 3
MHC: major histocompatibility complex
MSC: multisynthetase complex
Puro: puromycin
SRP: signal recognition particle
SSCR: signal sequence coding region
