# Supplementary figures and images for: p180 Promotes the Ribosome-Independent Localization of a Subset of mRNA to the Endoplasmic Reticulum
Source: PLoS Biol. 2012 May 29;10(5):e1001336. doi: 10.1371/journal.pbio.1001336 (PMC3362647; doi:10.1371/journal.pbio.1001336)

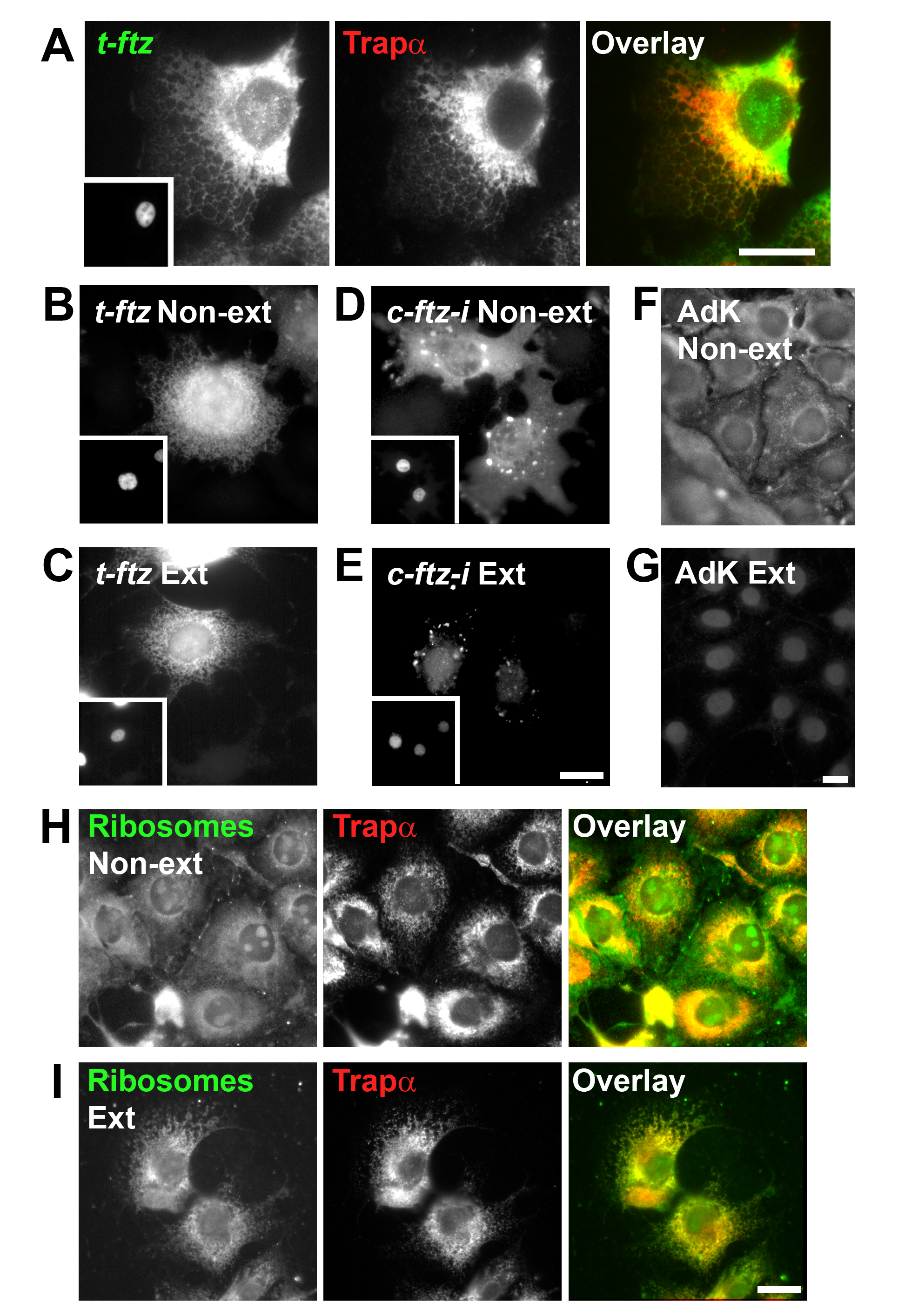

Supplement: Figure S1 — Visualization of ER-targeted mRNAs and ribosomes. (A) The nuclei of COS-7 cells were microinjected with t-ftz mRNA and Alexa488-conjugated 70 kD dextran (see inset) to label the microinjected compartment. The cell was incubated at 37°C for 2 h to allow for the nuclear export and targeting of the mRNA to the surface of the ER. A single cell is shown in (A) co-stained for ftz mRNA using specific FISH probes and for the ER marker Trapα by immunofluorescence. Note the extensive co-localization between the mRNA (green) and Trapα (red). Scale bar = 15 µm. (B–E) The nuclei of COS-7 cells were microinjected with either t-ftz (B–C) or c-ftz-i (D–E) mRNA and Alexa488 conjugated 70 kD dextran to label the microinjected compartment (insets). After incubating the cells at 37°C for 1 h, the cells were either directly fixed (“Non-ext”, B, D) or first extracted with digitonin (“Ext”, C, E) and then fixed. The cells were stained for ftz mRNA using specific FISH probes. Scale bar = 15 µm. (F–G) COS-7 cells were either directly fixed (“Non-ext”, F) or first extracted with digitonin (“Ext”, G) and then fixed and stained for Adenosine Kinase (“AdK”). Scale bar = 20 µm. (H–I) COS-7 cells were either directly fixed (“Non-ext”, H) or first extracted with digitonin and then fixed (“Ext”, I) and then fixed and stained for ribosome RPLP0 protein and the ER marker Trapα. Note the extensive co-localization between ribosomes (green) and Trapα (red) after extraction (I). Scale bar = 15 µm. (TIF) [file pbio.1001336.s001.tif]

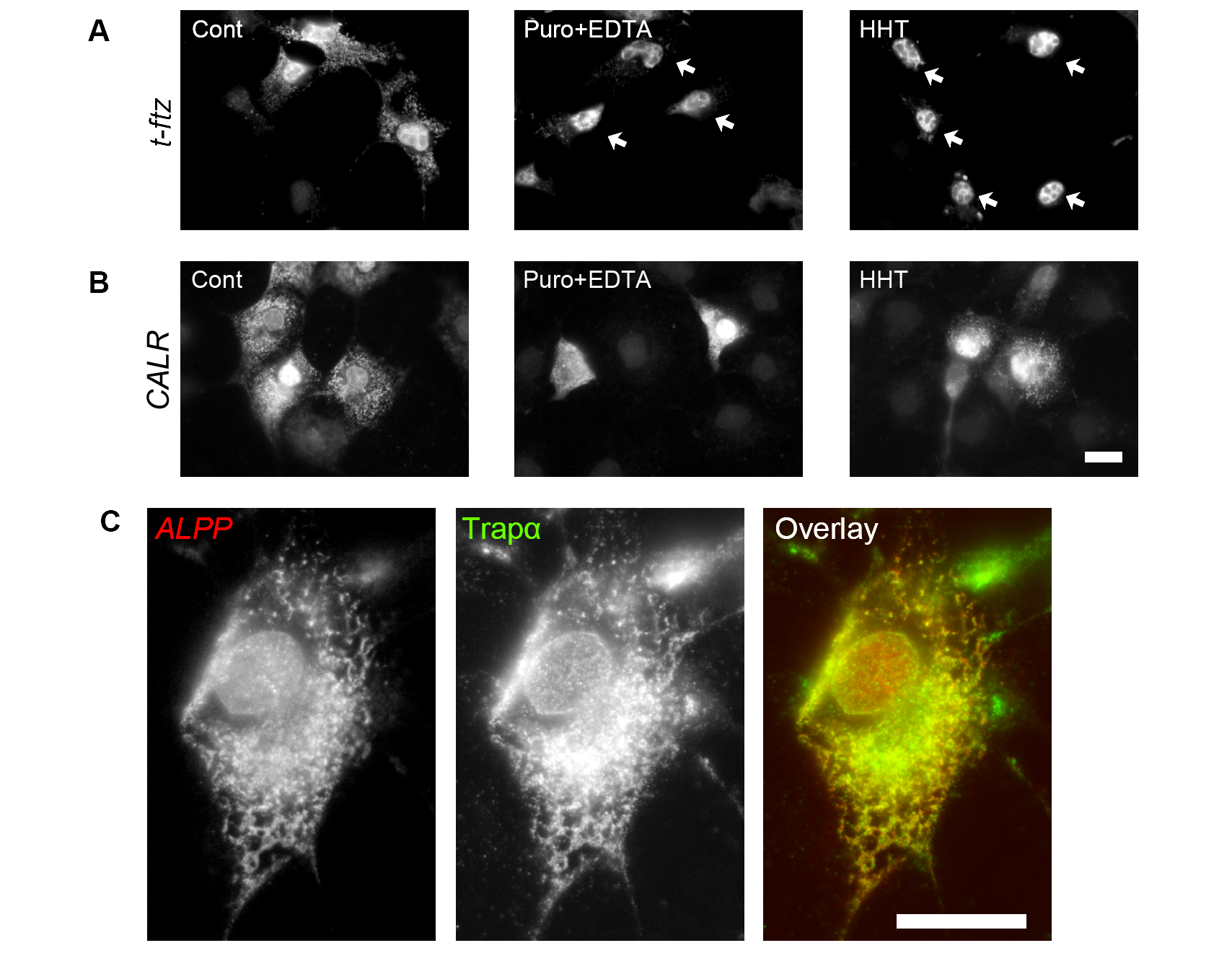

Supplement: Figure S2 — CALR mRNA, but not t-ftz mRNA, remains associated with the ER independently of ribosomes and translation. COS-7 cells were transfected with plasmids encoding either the t-ftz (A) or CALR (B) genes and were allowed to express mRNA for 18–24 h. The cells were then treated with control media (“Cont”), puromycin (“Puro”), or HHT for 30 min, and then extracted with digitonin alone or with 20 mM EDTA. Cells were then fixed, stained for mRNA using specific FISH probes, and imaged. Note that ER, but not nuclear, staining of t-ftz mRNA was lost after cells were treated with HHT or puromycin/EDTA (arrows). (C) COS-7 cells were transfected with plasmids encoding the ALPP gene and were allowed to express mRNA for 18–24 h. Cells were then treated with HHT for 30 min, then extracted with digitonin, fixed, and stained for ALPP by FISH and Trapα by immunofluorescence. Note the extensive co-localization of ALPP mRNA (red) and Trapα (green) in the overlay. (C) All scale bars = 20 µm. (TIF) [file pbio.1001336.s002.tif]

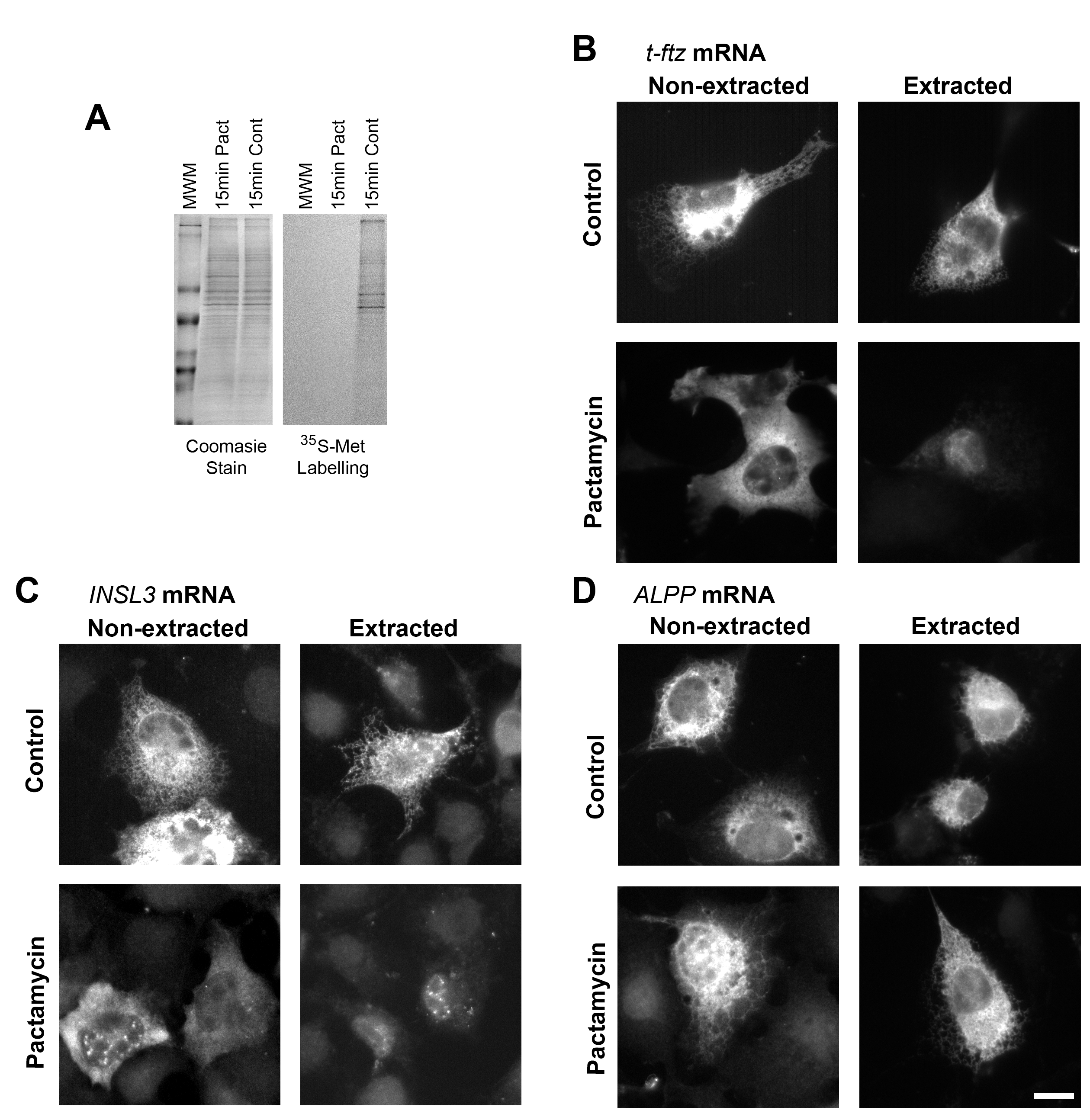

Supplement: Figure S3 — ALPP mRNA, but not t-ftz and INSL3 mRNAs, remains associated with the ER after cells were treated with pactamycin to disrupt the mRNA-ribosome association. (A) COS-7 cells were treated with control media (“Cont”) or pactamycin (“Pact”) for 15 min and then incubated in 35S-methionine to label newly synthesized proteins for an additional 15 min. Cell lysates were collected and separated by SDS-PAGE. Total proteins were visualized by Coomassie blue stain, and newly synthesized proteins were detected by autoradiography. Molecular weight markers are indicated on the left (“MWM”). (B–D) COS-7 cells were transfected with plasmids encoding either the t-ftz (B), INSL3 (C), or ALPP (D) genes and were allowed to express mRNA for 12–18 h. The cells were then treated with control media or pactamycin for 30 min, and then either directly fixed (“Non-extracted”) or first extracted with digitonin (“Extracted”) and then fixed. Cells were stained for mRNA using specific FISH probes and imaged. Scale bar = 15 µm. (TIF) [file pbio.1001336.s003.tif]

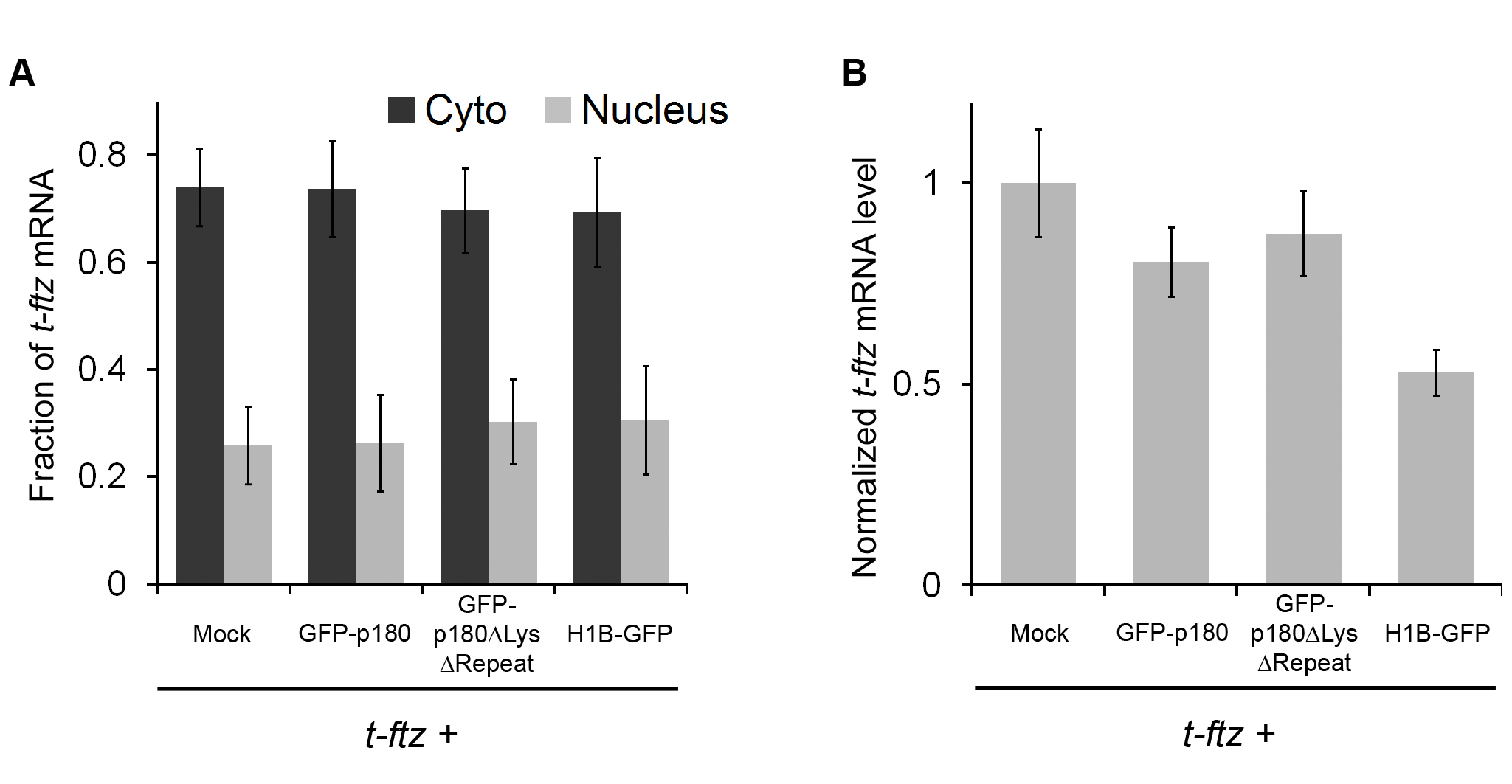

Supplement: Figure S4 — Nuclear export of t-ftz mRNA remains unchanged in co-transfected cells, but total t-ftz mRNA levels decrease in cells expressing H1B-GFP. (A–B) COS-7 cells were transfected with either plasmids containing t-ftz alone or in combination with plasmids containing GFP-p180, GFP-CLIMP63, or H1B-GFP. Cells were allowed to express for 18–24 h, fixed, and stained for t-ftz mRNA using specific FISH probes. Note that the cells were not extracted prior to fixation. (A) The fraction of t-ftz mRNA in the cytoplasm and nucleus in co-transfected cells. (B) The total level of t-ftz mRNA in the co-transfected cells, normalized to cells expressing t-ftz alone. Each bar consists of the average and standard deviation of 30–35 cells. (TIF) [file pbio.1001336.s004.tif]

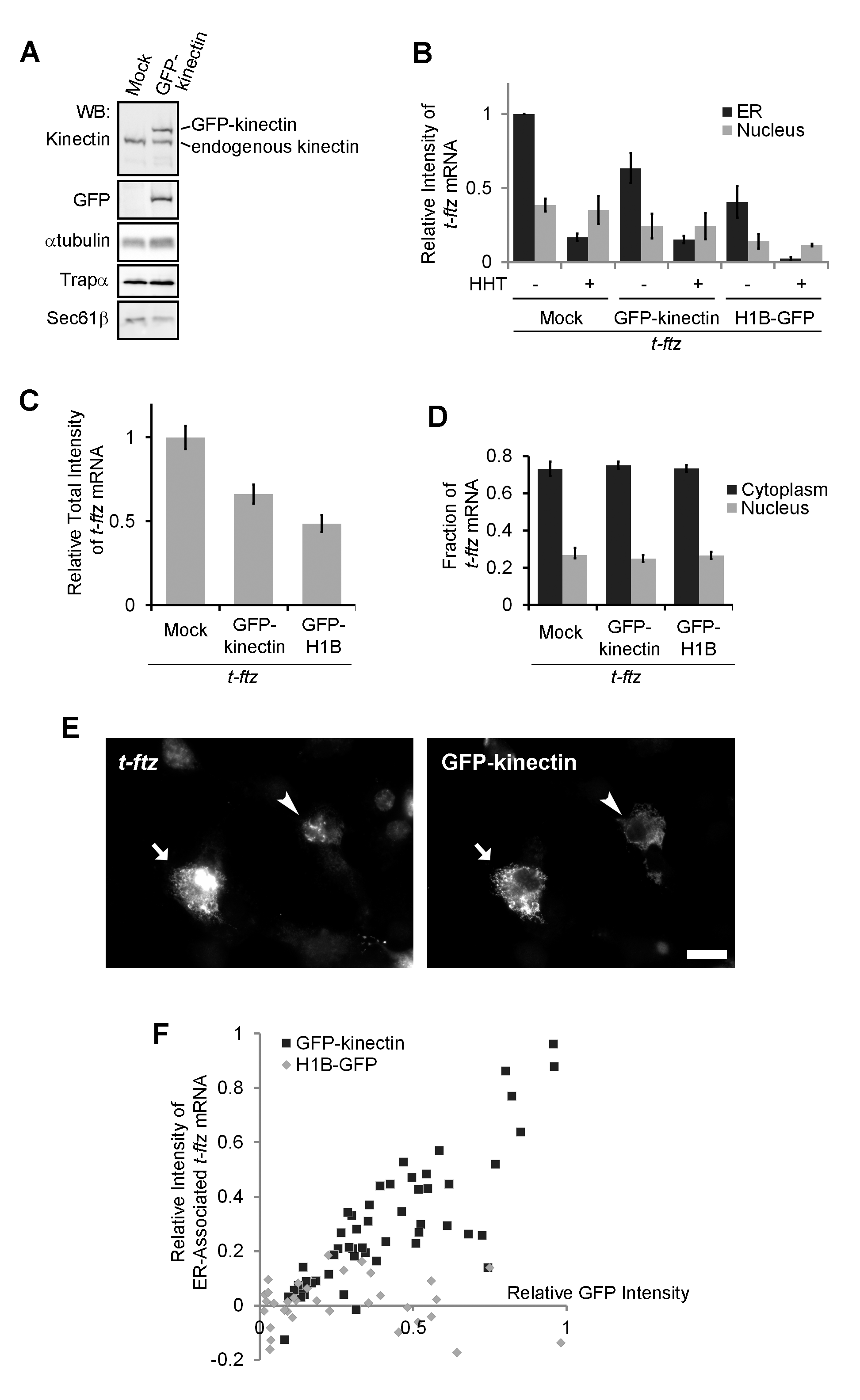

Supplement: Figure S5 — GFP-kinectin over-expression slightly enhances the ER-association of t-ftz mRNA after ribosome dissociation. (A) COS-7 cells were transfected without (“mock”) or with plasmids containing GFP-p180 and then lysed after 18–24 h. Cell lysates were separated by SDS-PAGE and immunoblotted for kinectin, GFP, αtubulin, and translocon components (Sec61β and Trapα). (B–F) COS-7 cells were transfected with plasmids containing t-ftz alone (“mock”) or in combination with GFP-kinectin or H1B-GFP. After 18–24 h cells were treated with control medium (B–D) or HHT for 30 min (B,E–F). Next, the cells were either first extracted with digitonin and then fixed to assess ER-association (B, E–F) or directly fixed to assess the total mRNA (C–D). After staining for t-ftz mRNA using specific FISH probes, cells were imaged. (B) The fluorescence intensity of t-ftz mRNA in the ER and nucleus in extracted cells. (C) The total level of t-ftz mRNA in unextracted cells. (D) The fraction of t-ftz mRNA in the cytoplasm and nucleus. (B–D) All data points are normalized to “mock” (cells expression t-ftz alone). Each bar represents the average and standard error of three independent experiments, each consisting of the average integrated intensity of 30 cells over background. (E) A single field of HHT-treated cells (30 min) that was imaged for t-ftz mRNA and GFP-kinectin. Scale bar = 20 µm. Note that t-ftz mRNA remains associated to the ER in cells with very high levels of GFP-kinectin (arrow), but not those with low levels (arrowhead). (F) For each HHT-treated cell the total level of ER-associated t-ftz FISH signal (normalized from the background (0), to the brightest cell in the experiment (1); y-axis) was plotted against total integrated GFP signal (normalized from the background (0), to the brightest cell in the experiment (1); x-axis). (TIF) [file pbio.1001336.s005.tif]

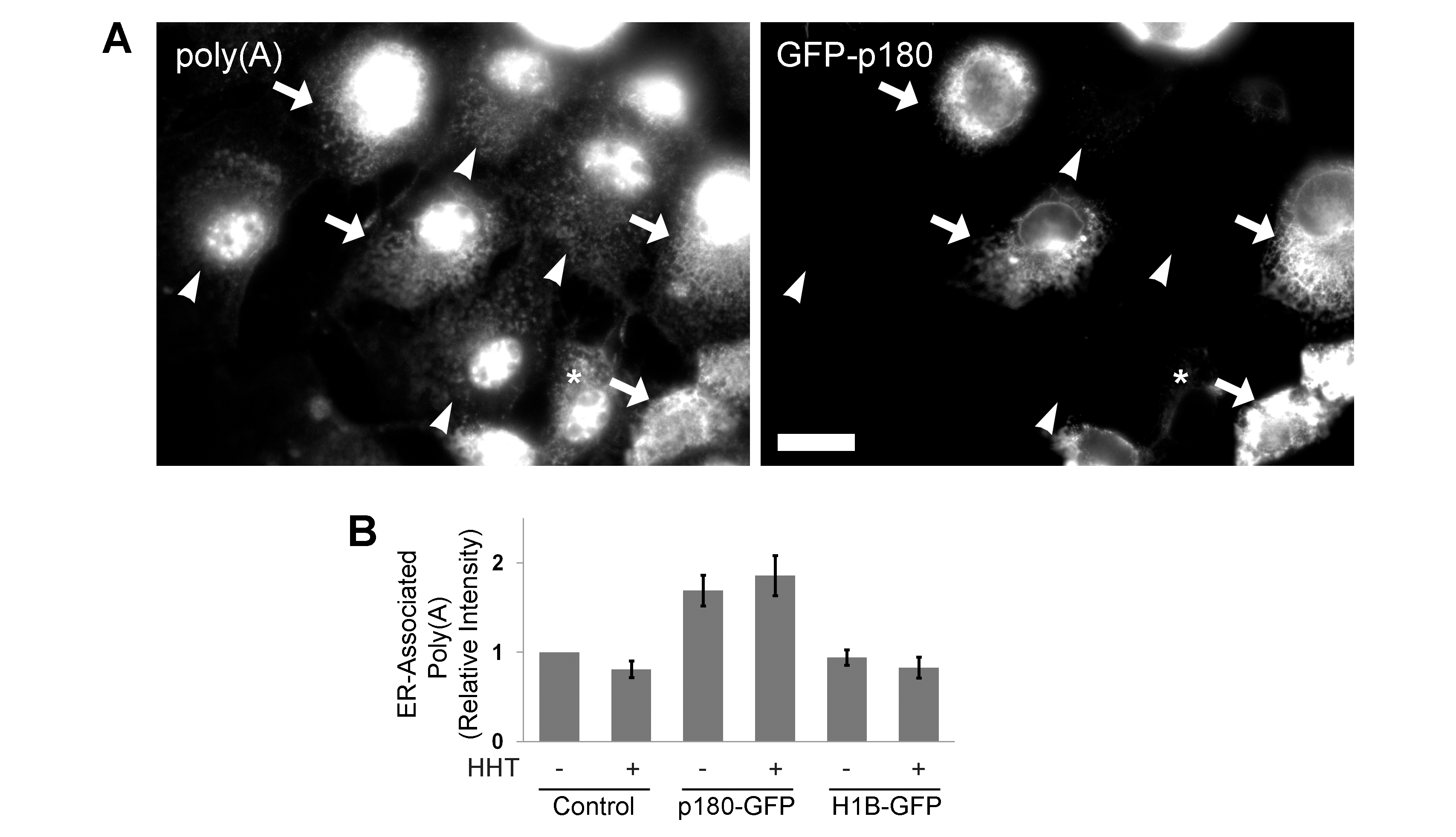

Supplement: Figure S6 — GFP-p180 over-expression enhances the ER-association of bulk poly(A) mRNA. COS-7 cells were transfected with either plasmids containing GFP-p180 or H1B-GFP and then fixed after 18–24 h. Cells were then treated with either control medium or HHT for 30 min to disassemble ribosomes, and then extracted, fixed, and stained poly(A) mRNA using poly(dT) FISH probes. (A) A single field of HHT-treated cells that was imaged for poly(A) mRNA and GFP. Cells expressing GFP-p180 are denoted by arrows, while untransfected cells are indicated by arrowheads. A cell with low GFP-p180 expression is denoted by an asterisk. Scale bar = 20 µm. The fluorescence intensity of mRNA in the ER was quantified (B). Each bar represents the average and standard error of three independent experiments, each consisting of the average integrated intensity of 50 cells over background. (TIF) [file pbio.1001336.s006.tif]

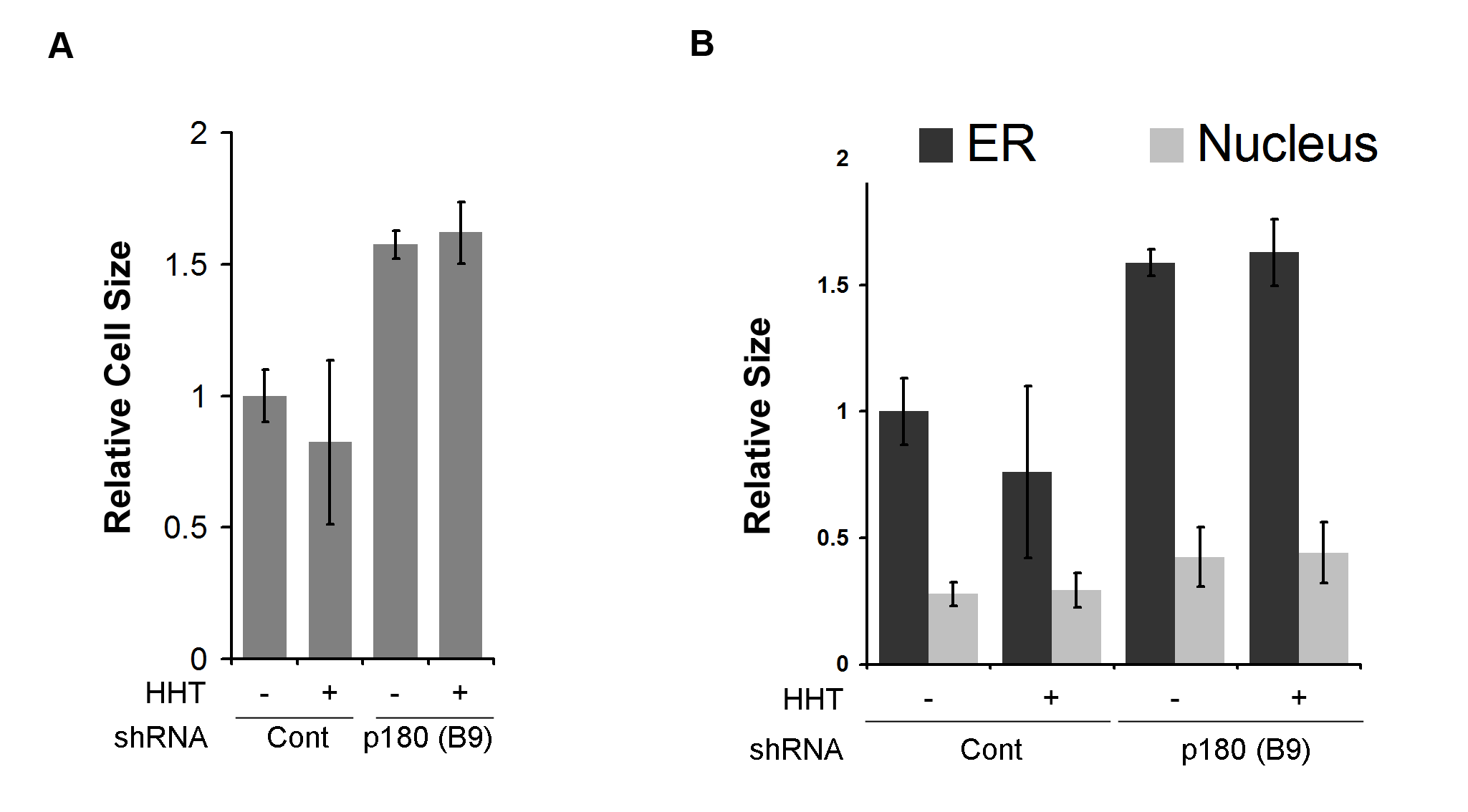

Supplement: Figure S7 — Depletion of p180 in U2OS cells increases cell size. The area in square pixels of the whole cell (A) or the ER and the nucleus (B) were measured in U2OS cells depleted of p180 with specific shRNAs or infected with control lentivirus and treated with control media or HHT for 30 min prior to digitonin extraction. All values were normalized to the size of either control cells (A) or ER (B). Each bar represents the average and standard error of four independent experiments, each consisting of the average from >30 cells. (TIF) [file pbio.1001336.s007.tif]
