# Supplementary material for: p180 Promotes the Ribosome-Independent Localization of a Subset of mRNA to the Endoplasmic Reticulum
Source: PLoS Biol. 2012 May 29;10(5):e1001336. doi: 10.1371/journal.pbio.1001336 (PMC3362647; doi:10.1371/journal.pbio.1001336)
Supplement: Table S1 — Proteins enriched in the ERMAP fraction (p>0.05). List of additional proteins (p>0.05) identified in the ERMAP fraction in addition to proteins listed in Table 1. Included in the table are the Entrez Gene ID, average number (“AVG”), and standard deviation (“STD”), of peptides from the analyses performed on three independent experiments. The p values were determined using a paired two-tailed Student t test. (RTF) [file pbio.1001336.s008.rtf]

Proteins	Gene ID	Rnase+	Rnase-	P Value	
		AVG	STD	AVG	STD		
HNRNP F	3185	4.3	1.3	1.5	1.2	0.05	
HNRNP M	4670	9.0	2.3	3.0	3.2	0.06	
Leucine-rich repeat-containing protein 59	55379	9.0	3.7	3.5	1.2	0.06	
Polypyrimidine tract-binding protein 1	5725	14.3	1.3	9.1	1.5	0.07	
SMC1a (structural maintenance of chromosomes 1A)	8243	5.3	0.0	3.8	0.0	0.07	
HNRNP H	3187	8.3	3.3	3.2	1.5	0.07	
RPL10A	4736	12.7	2.7	6.4	3.1	0.07	
Vigilin	3069	15.7	5.3	6.1	4.2	0.07	
Matrin-3	9782	5.0	0.0	3.6	0.0	0.07	
Hu-R/ELAV-like protein 1	1994	14.0	8.0	1.0	4.4	0.08	
Partner of Y14 and mago homolog	84305	4.7	0.0	3.5	0.0	0.08	
Developmentally-regulated gtp-binding protein 1	4733	8.0	0.0	6.1	0.0	0.08	
regulator of nonsense transcripts homolog	5976	36.7	17.0	11.0	10.4	0.09	
interleukin enhancer binding factor 2, 45kDa	3608	15.3	8.0	4.5	3.6	0.09	
DEAH box polypeptide 30	22907	3.7	0.7	2.1	1.2	0.09	
SRP receptor B	58477	6.0	1.0	3.6	1.7	0.10	
NOP2/Sun domain family, member 2	54888	13.0	3.7	6.1	4.6	0.10	
HNRNP K	3190	13.3	7.3	4.9	1.2	0.11	
HNRNP A1	3178	13.3	9.7	0.6	3.1	0.11	
DNA helicase Q1-like	5965	4.0	3.6	0.0	0.0	0.13	
HNRNP U-Like 1	11100	19.0	6.0	8.7	7.2	0.13	
MTHFD1L, methylenetetrahydrofolate dehydrogenase 1-like	25902	3.3	3.1	0.0	0.0	0.13	
Nucleolin	379633	49.7	15.6	27.7	12.9	0.13	
eIF2-gamma	1968	5.7	1.2	2.7	2.5	0.13	
DEAH box polypeptide 36	170506	7.3	2.3	2.3	4.0	0.14	
c1-tetrahydrofolate synthase cytoplasmic	4522	9.0	8.5	0.0	0.0	0.14	
Zinc finger CCCH type antiviral protein 1	56829	7.7	4.7	2.0	2.6	0.14	
cytoskeleton associated protein 5/ch-TOG	9793	12.0	11.5	0.0	0.0	0.15	
Signal recognition particle receptor alpha	6734	12.0	7.8	3.3	3.5	0.15	
Ubiquitin associated protein 2-like	9898	7.3	4.0	3.3	0.6	0.16	
Purine-rich element-binding protein B	5814	4.3	2.1	1.3	2.3	0.17	
Staufen 2	27067	3.3	3.5	0.0	0.0	0.18	
Heterogeneous nuclear ribonucleoprotein A3	220988	6.7	2.1	2.7	3.8	0.18	
AU-rich element RNA-binding protein 1	3184	10.7	3.1	7.0	3.0	0.21	
SMCHD1	23347	3.0	3.6	0.0	0.0	0.22	
SMC3 	9126	5.0	6.1	0.0	0.0	0.23	
DNA-dependent protein kinase, catalytic subunit	5591	10.7	12.2	0.7	1.2	0.23	
Purine-rich single-stranded DNA-binding protein alpha	5813	7.0	2.6	4.0	2.6	0.24	
Hu-B/elav-like protein 2	1993	4.3	3.8	1.0	1.7	0.24	
interleukin enhancer binding factor 3, 90kDa	3609	25.3	9.0	13.3	12.3	0.25	
CGI-99	51637	4.0	3.5	1.0	1.7	0.25	
RPLP0	6175	5.3	4.2	2.0	2.0	0.28	
poly-ADP ribose polymerase-1	142	4.7	4.2	1.3	2.3	0.29	
CNOT1, CCR4-NOT complex, subunit 1	23019	6.0	6.2	1.7	2.9	0.34	
Ribophorin 2	6185	10.7	5.0	7.2	7.8	0.41	
eIF4G1	1981	16.3	9.3	12.1	6.0	0.42	
Valyl-tRNA synthetase	7407	13.3	7.4	8.7	7.0	0.47	
Heterogeneous nuclear ribonucleoprotein B1	3181	8.0	7.2	4.3	3.8	0.48	
cold shock domain containing E1, RNA-binding	7812	5.0	7.0	1.7	2.9	0.49	
Chaperonin containing TCP1, subunit 5	22948	10.3	4.7	7.3	6.4	0.55	
Moloney leukemia virus 10, homolog	4343	14.0	9.2	10.7	7.6	0.65	
Ribophorin 1	6184	16.7	9.0	13.7	11.5	0.74	

Table S1. Proteins Enriched in the ERMAP Fraction (P>0.05).
List of additional proteins (P>0.05) identified in the ERMAP fraction in addition to proteins listed in Table 1. Included in the table are the Entrez Gene ID, average number (“AVG”), and standard deviation (“STD”), of peptides from the analyses performed on three independent experiments. P values were determined using a paired two tailed student T test.  
